# Coordinated Transcriptomic and Epigenetic Approach Reveals Molecular Features Underlying Natural Mating Ability in Captive Male Giant Pandas

**DOI:** 10.1002/ece3.72283

**Published:** 2025-10-12

**Authors:** Zheng Yan, Yinghu Lei, Pengpeng Zhao, Danhui Zhang, Jiena Shen, Guiquan Zhang, Rongping Wei, Mingyue Zhang, Dingzhen Liu

**Affiliations:** ^1^ Key Laboratory for Biodiversity and Ecological Engineering of Ministry of Education, Department of Ecology, College of Life Sciences Beijing Normal University Beijing China; ^2^ Research Center for the Qinling Giant Panda Shaanxi Rare Wildlife Rescue Base Xian China; ^3^ Key Laboratory of State Forestry and Grassland Administration on Conservation Biology of Rare Animals in the Giant Panda National Park, China Conservation and Research Center for the Giant Panda Dujiangyan China; ^4^ The Conservation of Endangered Wildlife Key Laboratory of Sichuan Province Chengdu Research Base of Giant Panda Breeding Chengdu China

**Keywords:** behavioral phenotype, captive breeding, conservation biology, epigenetic regulation, multi‐omics integration, reproductive behavior

## Abstract

Natural mating ability is a critical behavioral trait for the reproductive success of captive endangered mammals, and its loss often reflects declining adaptability and potential physiological dysfunctions. However, the underlying molecular regulatory mechanisms remain poorly understood. In this study, we integrated blood transcriptome and whole‐genome DNA methylation (whole‐genome bisulfite sequencing) data to systematically explore the molecular basis of natural mating ability differences in captive male giant pandas ( *Ailuropoda melanoleuca* ). A total of 21 male individuals, which were classified into either capable (with successful natural mating experience) or incapable (with repeated mating failure despite physical health) groups, were sampled from three breeding centers. RNA‐seq analysis identified key differentially expressed genes (DEGs) such as *ZPBP2*, enriched in functional pathways related to GnRH signaling, MAPK cascades, immune modulation, and olfactory perception. Whole‐genome bisulfite sequencing (WGBS) analysis revealed significant differences in CpG (CG) methylation density on the X chromosome, and identified promoter‐ and gene body‐associated differentially methylated regions (DMRs) that were inversely correlated with gene expression. Integrative analysis demonstrated a strong association between gene expression and DNA methylation, with the associated genes enriched in reproduction‐relevant pathways including axon guidance, cysteine and methionine metabolism, and apoptosis/autophagy. These findings suggest that DNA methylation may influence transcriptional activity involved in natural mating behavior. This multi‐omics approach provides valuable insights into the epigenetic regulation of complex reproductive phenotypes in endangered species and offers a theoretical basis for future applications in molecular marker–based individual selection and optimization of captive breeding programs, thereby contributing to wildlife conservation efforts.

## Graphical abstract

We investigated the molecular mechanisms underlying natural mating ability in captive male giant pandas using integrated transcriptomic and DNA methylation analyses. Our findings highlight key gene expression and epigenetic differences associated with reproductive behavior, offering novel insights for optimizing captive breeding and conservation strategies.

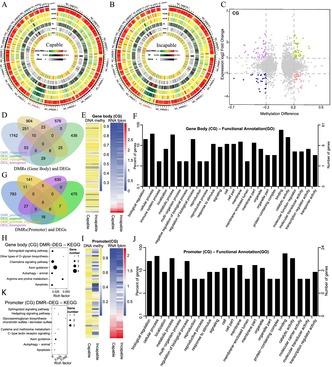

## 1 Introduction

**FIGURE 1:**
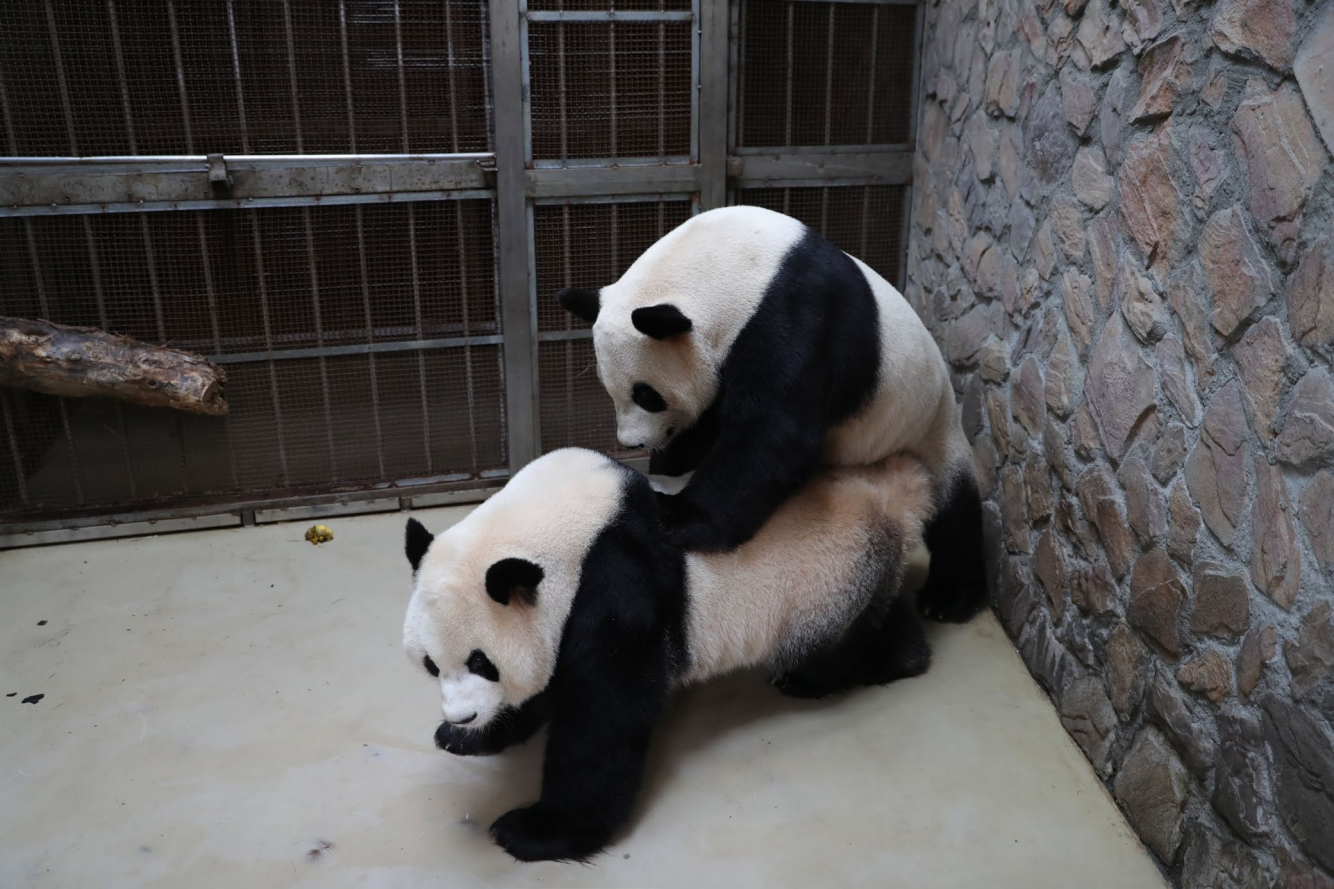
Captive giant pandas engaging in natural mating behavior at the Chengdu Research Base of Giant Panda Breeding (CRBGPB), Sichuan, China.

Natural mating ability is a critical trait for sustaining reproductive capacity and behavioral adaptability in captive large endangered mammals (Figure 1) (Holt and Pickard 1999; Li et al. 2017). Compared with assisted reproductive technologies such as artificial insemination, natural mating better preserves sexual selection, genetic diversity, and behavioral integrity, while minimizing the risks of behavioral abnormalities and reproductive failure associated with prolonged artificial intervention (AI) (Mason et al. 2007; Wildt et al. 2010). Moreover, excessive reliance on artificial insemination is often associated with lower birth rates (Li et al. 2017) and females subjected to artificial insemination may exhibit reduced maternal care or increased rejection of offspring (Li et al. 2022). In captivity, the loss of natural mating behavior is widely recognized as a manifestation of reduced adaptability, and restoring this ability has become a key focus of ex situ breeding programs (Price 1999; Shepherdson 1994). Impaired mating behavior may further erode genetic diversity and exacerbate population decline in endangered species (Dai et al. 2020; Martin‐Wintle et al. 2017; Seddon et al. 2014). Despite technological advances, many large endangered mammals continue to face challenges in achieving successful natural mating in captivity, including giant pandas ( *Ailuropoda melanoleuca* ) (Zhang, Zhang, et al. 2021), polar bears ( *Ursus maritimus* ) (Clubb and Mason 2007), cheetahs ( *Acinonyx jubatus* ) (Marker and O'Brien 1989), and clouded leopards ( *Neofelis nebulosa* ) (Tipkantha et al. 2017). For example, the general natural mating success rate of captive giant pandas remains below 50% from 1998 to 2018 (Zhang, Zhang, et al. 2021). Although considerable progress has been made in captive breeding, about 10%–30% of male giant pandas still lack sexual motivation or exhibit poor mating behavior, posing a major barrier to reproductive efficiency (Wei et al. 2012; Zhang et al. 2004). This causes nearly 20%–50% of cubs born each year to be bred by AI (D Liu, personal statistics). Although multiple external factors, including hormone levels, sperm quality, social experience, behavioral expression, mate choice, diet, and gut microbiota, have been linked to mating behavior (Martin‐Wintle et al. 2017, 2015; Martin et al. 2020; Yan et al. 2024, 2025; Zhang et al. 2022, 2023), substantial variation in mating ability among males persists even under controlled conditions, suggesting that intrinsic molecular mechanisms may play a decisive role.

Recent advances in high‐throughput omics have opened new avenues to investigate the molecular basis of natural mating behavior. Transcriptomic profiling, which captures genome‐wide gene expression patterns, has been widely applied to studies of reproductive behavior, spermatogenesis, and gonadal development (Chen et al. 2018; Green et al. 2018). For instance, transcriptome analyses in rodents, livestock, and non‐human primates have revealed that pathways such as gonadotropin‐releasing hormone (GnRH) signaling, mitogen‐activated protein kinase (MAPK) cascades, and sex hormone biosynthesis play crucial roles in regulating male reproductive capability (Adams 2005; Luo et al. 2022). However, transcriptional changes alone reflect transient gene activity and provide limited insight into upstream regulatory mechanisms. DNA methylation, one of the most stable and widespread epigenetic modifications in mammals, is known to regulate gene transcription and influence reproductive development, sexual behavior, and individual behavioral plasticity (Breton‐Larrivée et al. 2019; Luján et al. 2019; Zhang et al. 2024). Aberrant DNA methylation has been associated with reduced androgen levels (Mishra et al. 2010), disrupted reproductive organ function (Han et al. 2014), and even altered mate recognition in males (Dulac and Torello 2003; Holman et al. 2019; Hou et al. 2022). Therefore, integrating transcriptomic and epigenomic data has emerged as a powerful strategy to dissect the complex regulatory networks underlying behavioral phenotypes such as natural mating ability, which are orchestrated by multi‐layered molecular processes (Itai et al. 2023; McCabe et al. 2020).

To address the current gap in understanding the molecular mechanisms underlying natural mating ability in captive male giant pandas, we conducted an integrative analysis of blood‐derived transcriptomic and whole‐genome DNA methylation (WGBS) data. By comparing individuals with and without natural mating ability, we employed high‐throughput RNA sequencing and WGBS to systematically identify DEGs and DMRs. Through functional enrichment and integrative analysis, we constructed a regulatory network linking transcriptomic and epigenetic variation to reproductive behavior. Compared to single‐omics approaches, this multi‐omics strategy significantly enhances our ability to interpret complex behavioral phenotypes and uncovers the dynamic interplay between gene expression and epigenetic modifications (Santamarina‐Ojeda et al. 2023). These findings not only shed light on the epigenetic regulation of male reproductive function but also provide valuable molecular targets for improving captive breeding interventions and inform broader conservation strategies for endangered mammalian species.

## 2 Material and Methods

### 2.1 Ethics Statement

Blood samples used in this study were collected during the annual routine health examination. No giant panda was hurt, and they recovered well after the completion of sampling. The protocol of this study was approved by the Animal Welfare and Ethics Committee of Beijing Normal University (Approval number: CLS‐AWEC‐E‐2022‐002‐1).

### 2.2 Subjects, Blood Samples Collection, and Experimental Design

Male giant pandas that successfully mated with estrous females, completing mounting, insertion, and ejaculation, and whose mating resulted in the female giving birth to cubs, were classified as the capable group. Males that failed to mate successfully after multiple attempts with several estrous females during the mating season, showing no interest in females, exhibiting aggressive behavior toward females, or attempting to mount with incorrect posture or direction, were classified as the incapable group (Zhang et al. 2004).

All blood samples were collected during the giant panda mating season, from March to May each year. Blood samples were obtained from 7 captive giant pandas at the Research Center for the Qinling Giant Panda (RCQGP), Shaanxi Rare Wildlife Rescue Base (4 capable and 3 incapable individuals); 8 pandas from the Chengdu Research Base of Giant Panda Breeding (CRBGPB), Sichuan, China (3 capable and 5 incapable individuals); and 6 pandas from the Shenshuping Panda Base of the China Conservation and Research Center for the Giant Panda (CCRCGP), Wenchuan, Sichuan, China (4 capable and 2 incapable individuals). On the basis of sample availability and age, 18 individuals (9 capable and 9 incapable) with an average age of 13.83 ± 4.32 years were selected for transcriptome sequencing analysis. Additionally, transcriptome sequencing and analysis were performed separately for samples from RCQGP and CRBGPB, whereas samples from CCRCGP were excluded from separate analysis because of insufficient sample size. Subsequently, methylation analysis and integrative transcriptome–methylome analysis were conducted on the CRBGPB panda samples.

### 2.3 RNA Extraction and Library Construction

We extracted RNA using the PAXgene Blood RNA Kit (Qiagen, Germany) according to the instructions of the manufacturer. Subsequently, we used the Agilent 2100 bioanalyzer (Agilent Technologies, CA, USA) to determine the purity, concentration, and integrity of the RNA samples. The starting material for library construction was total RNA, which we enriched for mRNA with polyA tails using oligo (dT) magnetic beads. The resulting mRNA was then randomly fragmented using divalent cations in the Fragmentation Buffer. We used the fragmented mRNA as a template and random oligonucleotides as primers to synthesize the first strand of cDNA in the presence of M‐MuLV reverse transcriptase. We then degraded the RNA chain using RNase H and synthesized the second strand of cDNA using dNTPs as substrates in the presence of DNA polymerase I. The purified double‐stranded cDNA was subjected to end repair, A‐tailing, and ligation of sequencing adapters. We then used AMPure XP beads (Beckman Coulter, CA, USA) to select cDNA fragments of 370–420 bp, followed by PCR amplification and purification of the PCR products using AMPure XP beads. Finally, we obtained the library. After library construction, we first quantified the library using the Qubit 2.0 Fluorometer (Thermo Fisher Scientific, MA, USA), diluted it to a concentration of 1.5 ng/μL, and then checked the insert size of the library using the Agilent 2100 bioanalyzer. Once the insert size met the expected criteria, we used qRT‐PCR to accurately quantify the effective concentration of the library (with a library effective concentration above 1.5 nM) to ensure library quality.

### 2.4 Transcriptome Sequencing, Quality Control, Sequence Alignment, and Expression Analysis

After passing the quality control, we pooled different libraries on the basis of their effective concentration and the desired amount of data for sequencing on the Illumina platform NovaSeq 6000 (Illumina, CA, USA). The sequencing was performed according to the principle of Sequencing by Synthesis, generating paired‐end reads of 150 base pairs (Anslan et al. 2021). The raw sequencing data obtained contain a small number of reads with sequencing adapters or low sequencing quality. To ensure the quality and reliability of data analysis, we filtered the raw data. This included removing reads with adapters, eliminating reads containing ambiguous bases (N represents undetermined base information), and discarding low‐quality reads (reads with more than 50% of bases with a Phred quality score ≤ 5). At the same time, we calculated metrics such as Q20, Q30, and GC content for the clean data. The RNA sequencing results of the blood samples from 18 giant pandas showed that each panda obtained an average of 50,488,209 ± 88,10,726 raw reads. We got an average of 49,666,805 ± 8,874,177 clean reads after adapter trimming and removal of low‐quality reads. The Q20 value of the data from all individuals was 98.10% ± 1.02% (Table S1). All subsequent analyses were performed on the basis of these high‐quality, clean data.

We used the HISAT2 (v.2.0.5) software to align high‐quality sequences to the giant panda reference genome. The panda reference genome and annotation (ASM200744v3) were downloaded from the NCBI database (https://www.ncbi.nlm.nih.gov/assembly). The results of mapping the 18 short‐read libraries to the giant panda reference genome showed that, on average, 46,442,538 ± 90,10,461 high‐quality reads were successfully aligned, accounting for an average retention rate of 93.27% ± 0.15%. The average number of uniquely mapped reads per sample was 44,333,973 ± 9,087,671, representing 88.89% ± 2.46% of the total reads, indicating that the majority of paired reads were correctly aligned (Table S2). Comparison with the expressed regions (exon) revealed that 77.17% ± 4.14% of the mapped reads were within the exons (Table S3). These results indicate that most of the reads originated from mature mRNA sequences. To identify novel transcripts, unannotated transcriptional regions, and new genes, we employed StringTie software (v.1.3.3) (Pertea et al. 2015) for read assembly and compared the results with the original genome annotation. We employed the featureCounts (v.1.5.0) tool to calculate the read counts mapped to each gene. Subsequently, we calculated the fragments per kilobase of exon model per million mapped fragments (FPKM) for each gene on the basis of its length and the mapped read counts. This method takes into account both sequencing depth and gene length when estimating gene expression levels and is currently widely used (Zhao, Li, et al. 2021). We extracted genes related to mating behavior on the basis of the annotation results from the non‐redundant protein sequence database (NR database) maintained by the NCBI.

### 2.5 Gene Functional Analysis and Differential Expression Analysis of Transcriptome

We utilized the clusterProfiler software (v.3.8.1) to perform Gene Ontology (GO) functional enrichment analysis (Berardini et al. 2010) and Kyoto Encyclopedia of Genes and Genomes (KEGG) pathway enrichment analysis (Kanehisa et al. 2016) on sets of mating behavior‐related genes. The enrichment analysis was conducted on the basis of the hypergeometric distribution principle (Cao and Zhang 2014).

Pearson correlation coefficients (*R* ^2^) were calculated on the basis of the FPKM values of all genes in each sample to assess inter‐sample correlations. In this study, samples with *R* ^2^ greater than 0.8 were considered strongly correlated. Principal coordinates analysis (PCoA) and statistical tests were performed using R software (v4.1.0) with the vegan package (v2.5–7). Raw read counts were first normalized to correct for sequencing depth. Bray–Curtis distance matrices were then calculated on the basis of gene expression matrices, followed by PCoA to extract the first two principal coordinate axes (PC1 and PC2) along with their explained variances. Visualization was conducted using the ggplot2 package (v3.3–5), presenting sample distributions on the PC1–PC2 plane as scatter plots with 95% confidence ellipses to evaluate intra‐group variation concentration.

We utilized the clusterProfiler software (v.3.8.1) to conduct Gene Ontology (GO) functional enrichment analysis (Berardini et al. 2010) and Kyoto Encyclopedia of Genes and Genomes (KEGG) pathway enrichment analysis (Kanehisa et al. 2016) for the sets of mating behavior‐related genes. The enrichment analysis was performed on the basis of the hypergeometric distribution principle (Cao and Zhang 2014).

### 2.6 Methylation Sample Preparation, Sequencing Data Acquisition, and Preprocessing

For each sample, 100 ng of genomic DNA was mixed with 0.5 ng of unmethylated lambda DNA, then fragmented to 200–400 bp using a Covaris S220 ultrasonicator. Bisulfite conversion of unmethylated cytosines was performed using the EZ DNA Methylation‐Gold Kit. After library construction, quality was assessed by the Agilent 5400 system and qPCR. Qualified libraries were subjected to paired‐end sequencing on the Illumina NovaSeq 6000 platform.

Following quality control, libraries were pooled on the basis of effective concentration and target data volume requirements, and sequenced using 150 bp paired‐end reads on the Illumina platform. Illumina sequencing utilizes the Sequencing by Synthesis (SBS) principle, where fluorescently labeled dNTPs, DNA polymerase, and primers are incorporated in a flow cell; each incorporated base emits a fluorescent signal captured and converted to sequence data.

Raw sequencing data were first evaluated using FastQC (v0.11.8), followed by quality filtering with fastp (v0.23.1) to generate high‐quality clean data, which were re‐evaluated by FastQC. Clean bisulfite‐treated reads were aligned to the reference genome (NCBI accession GCF_002007445.2_ASM200744v3) using Bismark (v0.24.0) (Krueger and Andrews 2011). The reference genome was pre‐processed by C → T and G → A conversions and indexed with Bowtie2 (Langmead and Salzberg 2012). Similarly, converted reads were mapped, and the best unique alignments were selected and re‐mapped to the original genome to obtain methylation information at cytosine sites. PCR duplicates—reads mapped to the same genomic position—were removed, and sequencing depth and genome coverage were calculated. Methylation calls were extracted using bismark_methylation_extractor (‐‐no_overlap), and bisulfite conversion efficiency was estimated on the basis of the unmethylated cytosines in lambda DNA (Gao et al. 2015).

Sequencing alignment rates ranged from 84.45% to 86.29%, with unique mapping rates between 81.09% and 82.62%, and duplicate read rates from 26.3% to 30.22% (Table S4). Genomic sequencing depth across samples ranged from 15× to 18×, averaging ~16×; over 93% of bases had ≥ 1× coverage, ~90% ≥ 5×, and ~80% ≥ 10×, indicating high sequencing depth and coverage (Table S5). Methylation analysis showed average coverage depths between 6.6× and 7.6×, with CG methylation levels accounting for 78.94% to 80.62%, whereas CHG and CHH methylation levels were low, at 0.41%–0.48% and 0.41%–0.46%, respectively (Table S6). Specifically, CHG and CHH refer to non‐CG DNA methylation contexts, where H represents any nucleotide except G.

### 2.7 Methylation Level and Differential Methylation Analysis

Methylation significance at each cytosine site was assessed using a binomial test on the basis of the number of methylated reads (mC), total cytosine reads (mC+unmethylated C, umC), and non‐conversion rate (r). Sites with a false discovery rate (FDR)–adjusted *p*‐value < 0.05 were defined as methylated. To evaluate overall genome‐wide methylation levels, the genome was divided into 10 kb bins, and methylation levels (ML) were calculated for methylated sites within each bin.

DMRs were identified using the DSS software (v2.12.0) (Feng et al. 2014; Wu et al. 2015), which employs Gamma‐Poisson or Beta‐Binomial distributions to estimate dispersion parameters and uses shrinkage methods to improve detection power. DMRs were categorized according to their genomic locations as gene coding regions (transcription start site, TSS to transcription end site, TES) and promoter regions (2 kb upstream of TSS).

For functional annotation, DMR‐associated genes were subjected to Gene Ontology (GO) enrichment analysis using the R package GOseq (Young et al. 2010), adjusting for gene length bias, with significance defined as *p* < 0.05. KEGG pathway enrichment was performed with KOBAS software (v3.0) (Mao et al. 2005) to explore potential biological functions of DMR‐related genes.

Correlation of methylation levels among samples was evaluated to assess experimental reliability and sample selection suitability. Pearson correlation coefficients were calculated separately for CG, CHG, and CHH sequence contexts using methylation levels aggregated in 2 kb bins (Smallwood et al. 2014).

Comparative analyses between groups were conducted at multiple levels, including overall methylation levels, methylation distributions across genomic functional regions, and methylation patterns in upstream and downstream 2 kb regions of genes (Song et al. 2013). Circos plots were used to visualize genome‐wide methylation levels and differential patterns across sample groups (Krzywinski et al. 2009; Kulis et al. 2012).

To compare methylation distribution patterns in different genomic functional elements (promoters, exons, introns, repeats, CpG islands) and sequence contexts (CG, CHG, and CHH) between capable and incapable groups, functional regions were divided into 20 equal‐length bins. Average methylation levels of cytosines within each bin were calculated and plotted using dual y‐axes to separately display CG and non‐CG (CHG and CHH) methylation levels. Further, to investigate methylation differences around transcription regulatory regions (2 kb upstream of TSS, gene body, and 2 kb downstream of TES), these regions were segmented into 50 bins, and mean methylation levels per bin were calculated and visualized with dual y‐axes to highlight differences in CG and non‐CG methylation patterns.

### 2.8 Integration Analysis of DNA Methylation and Transcriptome

On the basis of the integration of whole‐genome DNA methylation and transcriptome data, this study systematically assessed their association using various visualization and statistical methods. First, at the chromosomal level, biological replicate samples were merged within each group, and methylation densities for CG, CHG, and CHH contexts, as well as transcriptome sequencing read densities, were calculated. Circos plots (v0.62‐1) were generated to display the spatial distribution across chromosomes of (1) CG, CHG, and CHH methylation densities; (2) transposable element (TE) coverage; (3) gene density; and (4) transcriptome read density (Wang et al. 2015).

Second, at the gene and flanking region level, all genes were classified into four expression categories—none (< 1), low (1 to first quartile), medium (first to third quartile), and high (> third quartile)—on the basis of FPKM values within each group (Xu et al. 2018). Specifically, for the Capable group, the 25th and 75th percentiles were 2.91 and 20.62, respectively; for the Incapable group, they were 3.02 and 22.12. Within the gene body and its ±2 kb flanking regions, each was divided into 50 equal bins, and average methylation levels of cytosines within each bin were computed and plotted for different methylation contexts across expression levels.

Third, genes were stratified into five quantiles (20%, 40%, 60%, 80%, and 100%) on the basis of methylation levels in promoters (2 kb upstream of TSS) and gene bodies. Expression distributions (log_2_FPKM+1) among groups were compared, and the correlation between methylation and gene expression was analyzed using boxplots and scatterplots.

For DMR‐associated gene analysis, differentially methylated regions were identified via Wald tests (threshold 0.2, p.threshold = 1e‐5), and their associated gene sets were intersected with DEGs (|log_2_FC| ≥ 1). Venn diagrams were constructed for four gene categories: hyper‐ and hypo‐methylated promoter‐ and gene body‐anchored genes overlapping with up‐ or downregulated DEGs (Yang et al. 2014). Overlapping genes were further analyzed by hierarchical clustering heatmaps and line plots depicting methylation dynamics across gene bodies and ±2 kb flanking regions.

Finally, GO enrichment was performed on overlapping gene sets using GOseq (Bioconductor 2.13) (Young et al. 2010), KEGG pathway enrichment using KOBAS (v2.0), and motif analysis using Homer (findMotifsGenome.pl v4.11) to identify the top 25 significant motifs of lengths 8, 10, 12, and 14 nucleotides in gene body and promoter regions. This comprehensive approach elucidates the molecular mechanisms underlying DNA methylation regulation of gene expression.

### 2.9 Statistical Analysis

To identify significantly DEGs in the blood transcriptome between the capable and incapable groups of giant pandas, we first calculated the log_2_‐transformed fold change (log_2_FoldChange) of gene expression between the two groups. Genes with an absolute log_2_FoldChange > 1 were selected for further analysis of expression differences. We then assessed the statistical significance of these differences and adjusted the *p* values using the Benjamini–Hochberg (BH) method to control the false discovery rate (FDR). On the basis of the DEG analysis results, we generated volcano plots to visualize the distribution of DEGs in each comparison group (Love et al. 2014). Volcano plots were generated to visualize the distribution of DEGs for each comparison. To pinpoint the most important genes distinguishing the capable and incapable groups, random forest analysis was performed using the R package randomForest (v.4.7–1.2), on the basis of gene FPKM values.

Pearson correlation analysis was conducted on DEGs to explore co‐expression patterns and identify potentially functionally related genes. Correlations with Pearson correlation coefficients > 0.5 or < −0.5 and *p* values < 0.05 were considered significant. Visualization of results was performed using the ggplot2 package in R (v.3.5.0) (Wickham 2011). Differences between groups in transcriptomic data were assessed using PERMANOVA on the basis of Bray–Curtis dissimilarity metrics. This analysis was implemented with the adonis2 function from the vegan package (v2.6–8) in R, with significance determined from 999 permutations.

DMRs were identified using the DSS (Dispersion Shrinkage for Sequencing data) software, specifically the DSS‐single model, which accounts for several key factors affecting methylation level estimation. To improve the accuracy and sensitivity of single‐site methylation estimates and DMR detection, DSS models spatial correlation among methylation sites by smoothing across neighboring CpGs, enhancing the detection of DMRs spanning long genomic regions. The model also incorporates sequencing read depth to stabilize methylation level estimates and improve statistical power. To address biological replicate variance, DSS uses a beta‐binomial distribution to model within‐group variability, reducing false positives. For datasets without biological replicates, DSS‐single applies a local smoothing approach, treating adjacent CpG sites as pseudo‐replicates, enabling approximate estimation of biological variance and supporting DMR detection under no‐replicate conditions.

## 3 Results

### 3.1 Gene Expression and Differentially Expressed Genes (DEGs) Associated With Natural Mating Ability in Male Giant Pandas

**FIGURE 2:**
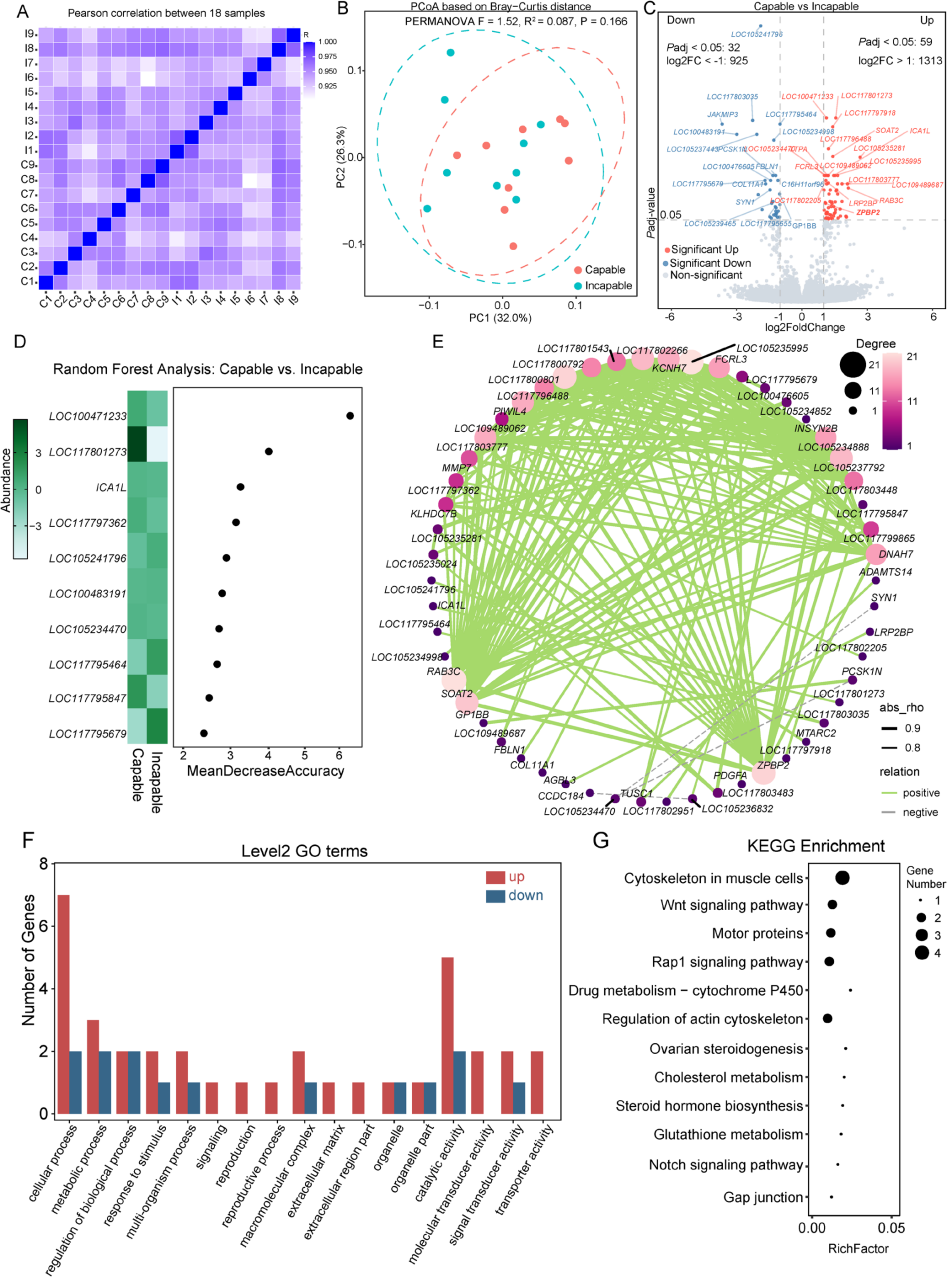
Differential gene expression and functional enrichment analyses of blood transcriptomes between the capable and incapable groups of captive male giant pandas. (A) Heatmap showing sample‐wise expression correlations on the basis of Pearson correlation coefficients. Values closer to 1 indicate stronger correlations; *R* ^2^ > 0.8 is considered highly correlated. (B) Principal coordinate analysis (PCoA) on the basis of Bray–Curtis dissimilarity. Each dot represents a sample, with colors indicating the Capable group (red) and Incapable group (blue). (C) Volcano plot of differentially expressed genes (DEGs) identified using DESeq2. Genes with |log_2_(FoldChange)| > 1 and adjusted *p* ≤ 0.05 were considered significant. (D) Random forest analysis identifying key feature genes distinguishing between Capable and Incapable groups. Genes with the highest Mean Decrease Accuracy are shown, along with their relative abundance in both groups. (E) Co‐expression network constructed on the basis of Pearson correlation of DEGs. Only gene pairs with *p* < 0.05 and either strong positive (*r* > 0.8) or negative (*r* < −0.7) correlations were retained. Line color and type indicate correlation direction; line thickness represents correlation strength; and node size and color depth reflect node degree. (F) GO enrichment analysis of DEGs. Red bars indicate GO terms enriched with more upregulated genes in the Capable group, whereas blue bars indicate enrichment in the Incapable group. (G) KEGG pathway enrichment of DEGs. The *x*‐axis shows the rich factor, point size reflects the number of involved genes, and color indicates the significance of enrichment.

A total of 33,286 genes, including 1954 novel genes, were identified in the blood transcriptomes of captive male giant pandas and used for gene expression quantification. Correlation analysis of FPKM values among samples from the naturally mating‐capable group (Capable) and the mating‐incapable group (Incapable) revealed strong intra‐group correlations (*R* ^2^ > 0.9; Figure 2A). Principal coordinate analysis (PCoA) on the basis of Bray–Curtis dissimilarity showed no significant separation between the two groups (*F* = 1.52, *R* ^2^ = 0.087, *p* = 0.166; PERMANOVA; Figure 2B). Consistent results were obtained when samples from two breeding bases—CRBGPB in Sichuan and RCQGP in Qinling—were analyzed separately (Figure S1).

Nevertheless, 91 DEGs were identified between the Capable and Incapable groups using the threshold of |log_2_(FoldChange)| > 1 and adjusted *p* ≤ 0.05. Of these, 32 genes were significantly downregulated and 59 were upregulated in the Capable group (Figure 2C), including key genes such as *LOC117800792*, *ZPBP2*, *DNAH7*, and *PIWIL4*. Random forest analysis identified the top 10 features contributing to group classification on the basis of the highest Mean Decrease Accuracy values. Excluding pseudogenes, notable contributors included *LOC117795464*, *LOC100483191*, *ICA1L*, *LOC105234470*, *LOC117795679*, *LOC117797362*, and *LOC117795847* (Figure 2D).

Co‐expression analysis on the basis of DEGs revealed five hub genes with the highest degree values: *LOC105235995*, *RAB3C*, *LOC117800801*, *ZPBP2*, and *SOAT2* (Figure 2E). GO enrichment analysis of genes upregulated in the Capable group highlighted several terms related to reproductive and signaling functions, including *reproductive process*, *reproduction*, *multi‐organism process*, *signal transducer activity*, and *signaling* (Figure 2F), pointing to an association with reproductive and signaling processes. Broader biological processes such as *response to stimulus*, *metabolic process*, and *cellular process* were also enriched, indicating elevated physiological responsiveness and metabolic activity.

Notably, *ZPBP2* (zona pellucida binding protein 2), a gene annotated in the reproductive GO terms, encodes a sperm protein involved in binding to the egg zona pellucida and contains a conserved PF07354 domain, critical for sperm–egg recognition. KEGG pathway analysis of DEGs further revealed enrichment in pathways relevant to reproduction and steroidogenesis, including *steroid hormone biosynthesis*, *ovarian steroidogenesis*, and *cholesterol metabolism* (Figure 2G). Enrichment of the *Wnt signaling pathway* and *Notch signaling pathway* also suggests potential involvement of testicular development and spermatogenesis (Figure 2G).

By comparing with the NR database, we identified a total of 320 genes directly related to mating behavior in male giant pandas, encompassing six functional categories: androgen signaling, GnRH signaling, sperm quality (including spermatogenesis, motility, binding, and fertilization), olfactory receptors, dopamine signaling, and germ cell development. Among these, GnRH pathway–related genes ranked within the top 20 by expression level (FPKM). Protein–protein interaction network analysis further highlighted 10 core proteins—RAF1, MAP2K1/2, MAP3K1, the RAS family, SOS1, and PIK3CA/D—indicating their central roles in key signaling pathways that regulate mating behavior (Figures S2–S4 and Tables S7 and S8, Supporting Information Results).

### 3.2 WGBS Analysis

**FIGURE 3:**
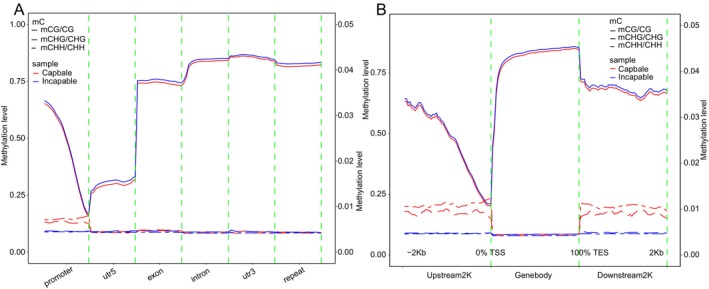
DNA methylation level distribution across genomic features and gene‐flanking regions in the capable and incapable groups. (A) Average methylation levels across different genomic features under three sequence contexts. For each functional element (including promoter, exon, intron, repeat, CGI, CGI shore, CGI shelves, and open sea), the regions were divided into 20 equal‐length bins, and the mean methylation level of cytosine sites within each bin was calculated. (B) Methylation level distribution upstream and downstream of genes under the three sequence contexts. The transcription start site upstream 2 kb (TSS upstream), gene body, and transcription end site downstream 2 kb (TES downstream) were divided into 50 bins, and the average methylation level of cytosines in each bin was calculated. The left y‐axis represents methylation levels in the CG context, and the right *y*‐axis represents methylation levels in non‐CG contexts (CHG and CHH). Line colors indicate different groups, and line styles represent different sequence contexts.

We performed WGBS on male giant pandas from the CRBGPB breeding unit in Sichuan. The sequencing data were of high quality, with raw read numbers ranging from approximately 236–275 million, and the proportion of clean reads after filtering ranged from 86.85% to 87.92%. Quality scores were high (Q20 > 96.7%, Q30 > 90.3%), GC content remained stable (21.3%–22.1%), and bisulfite conversion rates were consistently above 99.6% (Table S9). CG methylation levels and densities were high and stable across all samples, whereas CHG and CHH methylation levels and densities were substantially lower and exhibited greater variability between samples and across genomic bins (Table S10). Among the three sequence contexts, methylation was predominantly concentrated in CG sites, with CG methylation density reaching up to 99.17%, in contrast to maximum densities of only 0.25% and 0.21% in CHG and CHH contexts, respectively, reflecting the typical methylation pattern of mammalian genomes (Table S10). Within genomic functional regions, CG methylation levels were markedly higher in gene body regions such as exons and introns, whereas promoter and untranslated region (UTR) regions exhibited significant hypomethylation (Figure 3A). Although CHG and CHH methylation levels were globally low, slight differences between the two groups were observed in promoter and 5′ UTR regions. CG methylation exhibited a typical bimodal distribution pattern, characterized by decreased methylation near transcription start sites (TSS), elevated levels across gene bodies, and a subsequent decline near transcription end sites (TES). Notably, CHG and CHH methylation levels in upstream and downstream gene regions were consistently higher in the capable group compared to the incapable group (Figure 3B).

### 3.3 DMR Analysis

**FIGURE 4:**
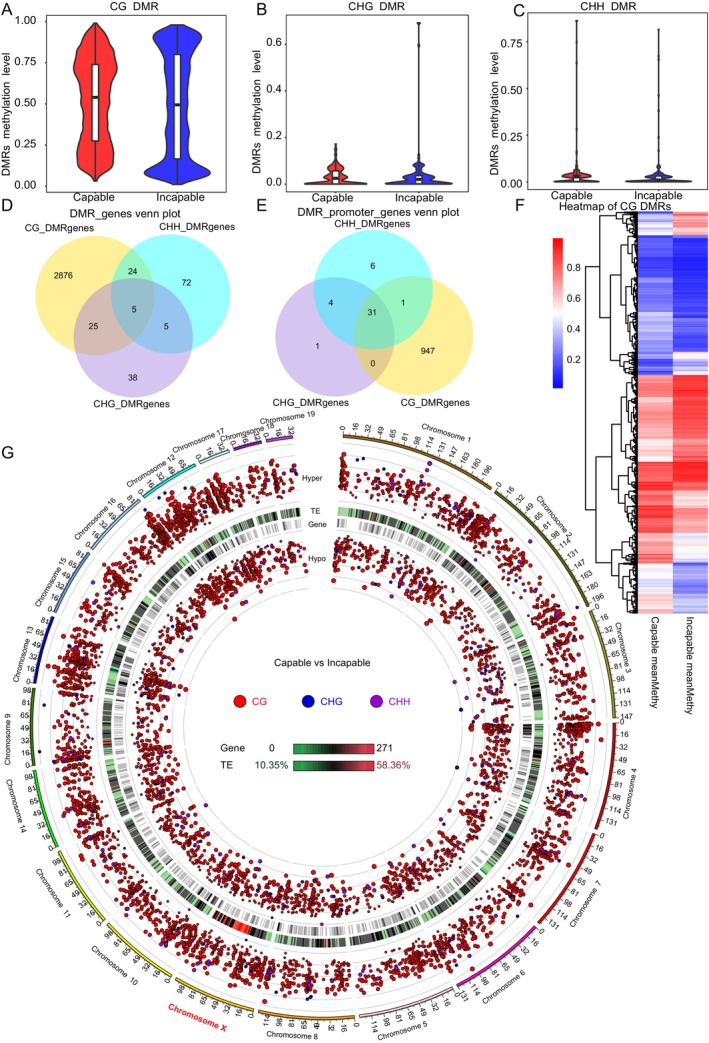
Distribution of DMR methylation levels and genomic localization features across different sequence contexts. (A–C) Violin plots showing the distribution of DNA methylation levels in differentially methylated regions (DMRs) identified in CG (A), CHG (B), and CHH (C) sequence contexts. The *x*‐axis represents the comparison groups, whereas the *y*‐axis indicates the methylation level. Each violin plot illustrates the overall distribution, with an embedded boxplot indicating the median and interquartile range. The shape of the violin reflects the kernel density estimation of methylation values across DMRs. (D) Venn diagram showing the overlap of genes anchored by DMRs within gene body regions across CG, CHG, and CHH contexts. (E) Venn diagram illustrating the number of genes associated with promoter‐region DMRs across the three sequence contexts. (F) Heatmap clustering on the basis of CG‐context DMR methylation levels. The *x*‐axis represents sample groups, and the y‐axis shows hierarchical clustering of DMRs by methylation level. Color gradients from blue to red denote increasing methylation levels. CHG and CHH context heatmaps are shown in Figure S6. (G) Circos plot showing the genome‐wide distribution of DMRs across CG, CHG, and CHH contexts. From the outermost to innermost circles: (1) Log_5_(|areaStat|) of hypermethylated regions (Hyper‐DMRs), where dot height indicates the magnitude of difference; CG‐DMRs are marked in red, CHG in blue, and CHH in purple. (2) Heatmap of transposable element (TE) content, with color intensity representing TE enrichment (see legend for scale). (3) Gene density heatmap, with color indicating gene abundance. (4) Log_5_(|areaStat|) values of hypomethylated regions (Hypo‐DMRs), where inner‐facing, higher dots indicate more pronounced hypomethylation differences; color coding as in the outer circle.

In the analysis of DMRs, CG methylation levels exhibited high consistency among all samples (Pearson's *R* ^2^ > 0.8), whereas the methylation levels in CHG and CHH contexts showed low correlations, with *R* ^2^ values not exceeding 0.1 and 0.12, respectively (Figure S5). The methylation differences between the Capable and Incapable groups were predominantly observed in the CG sequence context, which accounts for over 80% of the total DNA methylation (Figures S6–S8, Table S10). A total of 6656 DMRs were identified between the two groups, including 6321 CG‐type DMRs. However, the overall methylation level distributions across CG, CHG, and CHH contexts did not show significant differences between groups (Figure 4A–C). In the CG context, most DMR‐associated genes were anchored in the gene body regions (*n* = 2930), whereas only five genes were simultaneously annotated in all three contexts (CG, CHG, and CHH) (Figure 4D). Similarly, in the promoter regions, the CG context also yielded the highest number of DMR‐associated genes (*n* = 979), with 31 genes overlapping across all three sequence contexts (Figure 4E). Clustering heatmaps on the basis of CG‐context DMR methylation levels clearly distinguished Capable and Incapable individuals (Figure 4F), whereas CHG and CHH contexts, because of their globally low methylation levels, did not reveal obvious intergroup differences (Figure S9). Among all identified DMRs, 76 regions showed absolute methylation differences greater than 0.5 between groups, including 28 hypermethylated regions (all in CG context) and 48 hypomethylated regions (mostly CG, with 2 in CHG) in the Capable group (Figure 4G). In total, DMRs mapped to 3045 genes across various genic elements such as exons, introns, and UTRs (Table S11), and 990 genes were associated with DMRs located in promoter regions (Table S12).

### 3.4 Functional Enrichment Analysis of DMR‐Associated Genes

**FIGURE 5:**
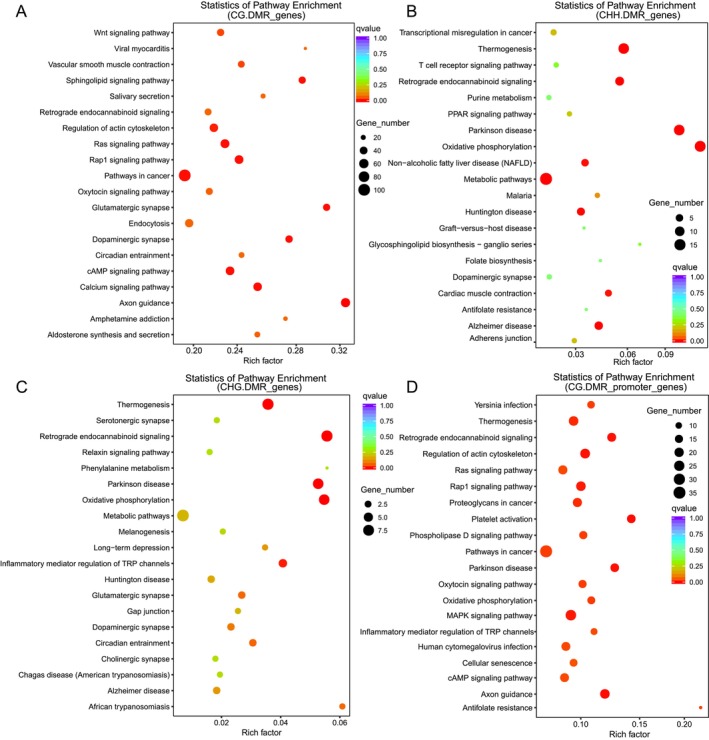
KEGG pathway enrichment analysis of DMR‐associated genes. (A) Dot plot showing KEGG pathway enrichment for DMR‐associated genes in the CG sequence context. (B) KEGG enrichment results for genes associated with CHH‐context DMRs. (C) KEGG enrichment for CHG‐context DMR‐associated genes. (D) KEGG pathway enrichment for genes with DMRs located in promoter regions. The *y*‐axis denotes the names of enriched pathways, and the *x*‐axis indicates the rich factor, representing the degree of enrichment. Dot size corresponds to the number of DMR‐associated genes involved in each pathway, whereas the color gradient reflects the adjusted Q‐value, with darker colors indicating higher statistical significance.

On the basis of the overlap between DMRs and genomic functional regions, we performed KEGG pathway enrichment analysis for genes associated with DMRs located in gene body regions. In the CG sequence context, DMR‐associated genes were significantly enriched (*p* < 0.05) in several signaling pathways, including axon guidance, calcium signaling pathway, glutamatergic synapse, Rap1 signaling pathway, Ras signaling pathway, Wnt signaling pathway, and regulation of actin cytoskeleton (Figure 5A). Further stratified analysis revealed that in the Capable group, hypermethylated (Hyper) DMR genes were mainly enriched in axon guidance, regulation of actin cytoskeleton, and Rap1 signaling pathway (Figure S10A), whereas hypomethylated (Hypo) DMR genes were significantly enriched in pathways in cancer, dopaminergic synapse, cholinergic synapse, circadian entrainment, Ras signaling pathway, phospholipase D signaling pathway, and calcium signaling pathway (Figure S10B).

In the CHH sequence context, DMR‐associated genes were significantly enriched in oxidative phosphorylation, Parkinson disease, thermogenesis, retrograde endocannabinoid signaling, metabolic pathways, Alzheimer disease, Huntington disease, non‐alcoholic fatty liver disease (NAFLD), and cardiac muscle contraction (Figure 5B). For the CHG context, enriched pathways included retrograde endocannabinoid signaling, oxidative phosphorylation, Parkinson disease, and thermogenesis (Figure 5C). Additionally, genes with promoter‐region DMRs were significantly enriched in the MAPK signaling pathway, regulation of actin cytoskeleton, Rap1 signaling pathway, and cAMP signaling pathway (Figure 5D).

### 3.5 Integrated Analysis of DNA Methylation and Transcriptomic Profiles

**FIGURE 6:**
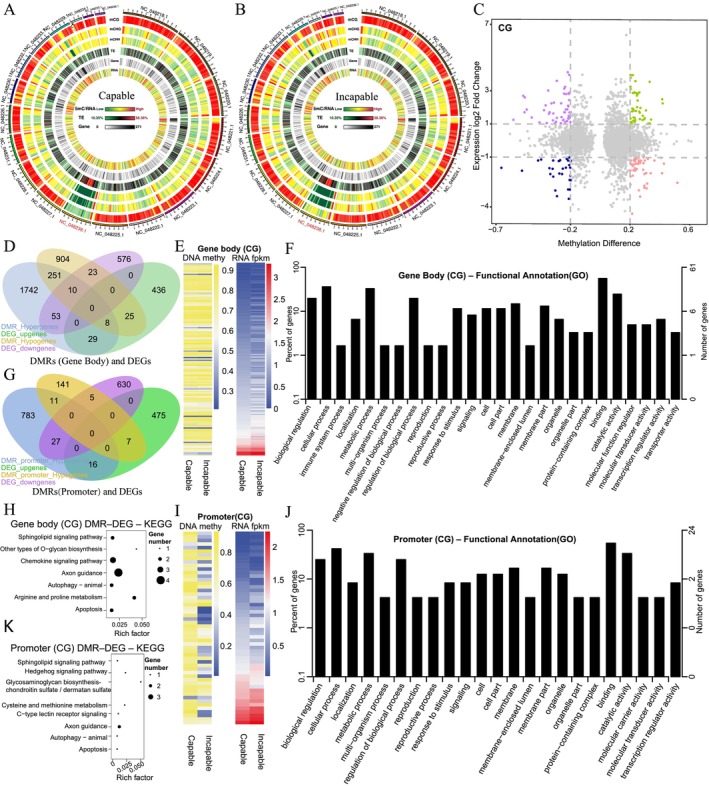
Integrated analysis of DNA methylation and transcriptomic profiles in Capable and Incapable male giant pandas to identify key regulatory genes. (A, B) Circos plots showing chromosome‐wide distribution of methylation and transcriptomic data for the Capable (A) and Incapable (B) groups. From outermost to innermost tracks: CG, CHG, and CHH methylation density heatmaps; TE (transposable element) density heatmap; gene density heatmap; and RNA‐seq read density heatmap. Color scales: Green to red (methylation and RNA‐seq read density), green to black to red (TE density), and white to black (gene density). TE density reflects the proportion of repeat elements in each bin; gene and read density represent the number of genes and mapped reads per bin, respectively. (C) Correlation plot of differential methylation levels versus differential gene expression. The *x*‐axis shows methylation level differences of DMRs; the *y*‐axis shows the corresponding log_2_(Fold change) of gene expression. Thresholds: |methylation difference| > 0.2, |log_2_FC| > 1. Genes in the top left quadrant indicate hypermethylation with downregulated expression. (D, G) Venn diagrams illustrating the overlap between DMR‐associated genes and differentially expressed genes (DEGs) in gene body (D) and promoter (G) regions. Categories include hypermethylated and hypomethylated genes in both regions (DMR_Hypergenes, DMR_Hypogenes) and upregulated DEGs (DEG_upgenes). (E, I) Heatmaps of hierarchical clustering for the intersected genes from (D) and (G), showing methylation levels and expression levels (log_2_(FPKM+1)) in gene body (E) and promoter (I) regions after Z‐score normalization. (F, J) GO enrichment bar plots for CG‐context DMR‐associated DEGs in gene body (F) and promoter (J) regions. (H, K) KEGG pathway enrichment scatterplots for the same gene sets in gene body (H) and promoter (K) regions. The *x*‐axis shows the rich factor (ratio of enriched genes to total annotated genes in the pathway), dot size indicates the number of intersected genes, and color gradient reflects adjusted *Q*‐values.

Overall, the chromosomal distribution patterns of DNA methylation levels and gene density were comparable between the Capable and Incapable groups. However, a marked difference in CG methylation density was observed on the X chromosome (NC_048238.1) (Figure 6A,B). By integrating methylome and transcriptome datasets, a total of 3094 overlapping genes were identified between DEGs and genes associated with DMRs (Table S13). Among these, 47 genes showed an inverse relationship with increased methylation and decreased expression, whereas 45 genes exhibited decreased methylation and increased expression (Figure 6C), on the basis of a differential methylation threshold of 0.2 and a |log_2_(Fold Change)| threshold of > 1. In the gene body regions, 70 and 96 genes exhibited positive and negative correlations between DMRs and DEGs, respectively, whereas in the promoter regions, 21 and 34 genes showed such associations (Figure 6D,G). Correlation and clustering analyses of CG‐context DMRs with gene expression revealed that promoter methylation differences between groups were more pronounced than those observed in gene body regions (Figure 6E,I). Functional enrichment of intersected genes with gene body methylation indicated significant GO terms such as “protein metabolic process”, “cellular biosynthetic process”, and “regulation of nitrogen compound metabolic process” (Figure 6F and Table S14), and KEGG pathways including Axon guidance, Chemokine signaling pathway, Sphingolipid signaling pathway, Apoptosis/Autophagy, Arginine and proline metabolism, and O‐glycan biosynthesis (Figure 6H and Table S15). For genes with promoter‐associated DMRs, GO enrichment was observed for “protein metabolic process”, “nucleoside phosphate binding”, and “regulation of biosynthetic process” (Figure 6J and Table S16), whereas KEGG pathways were enriched in Glycosaminoglycan biosynthesis, C‐type lectin receptor signaling, Lysosome, Axon guidance, Hedgehog signaling, Cysteine and methionine metabolism, Inositol phosphate signaling, and VEGF signaling (Figure 6K and Table S17).

## 4 Discussion

In this study, we integrated transcriptomic and WGBS data to investigate the molecular basis underlying the natural mating ability of captive male giant pandas. Specifically, our study found that although the global gene expression profiles showed no significant separation between the Capable and Incapable groups, differential expression analysis revealed a subset of genes associated with spermatogenesis, sperm‐egg recognition, and testis development (e.g., *ZPBP2*, *PIWIL4*, and *DNAH7*). This suggests that natural mating ability may be regulated by a small number of key functional genes. Furthermore, WGBS analysis demonstrated typical mammalian DNA methylation patterns, with high and stable CG methylation and low non‐CG methylation (CHG/CHH), but revealed subtle group‐level differences, particularly in the CHG and CHH contexts around gene flanking regions. Importantly, DMRs were predominantly observed in the CG context and were significantly enriched in genes involved in axon guidance, reproductive signaling, and hormone‐related pathways. Integrative analysis of methylation and gene expression further identified genes exhibiting classical methylation‐expression anticorrelation patterns, particularly within promoter regions, highlighting potential epigenetic regulation of transcriptional activity. Additionally, the differential methylation observed on the X chromosome implies possible involvement of sex chromosome‐linked regulatory elements. Together, these findings indicate that the natural mating capability in male giant pandas may be influenced by coordinated transcriptional and epigenetic regulation of genes associated with reproductive function, neural signaling, and cellular communication.

Our transcriptomic profiling of captive male giant pandas with distinct natural mating abilities revealed a subset of DEGs that are closely associated with reproductive physiology, neuroendocrine signaling, and cellular regulation. Notably, *ZPBP2*, a gene encoding zona pellucida‐binding protein 2, was significantly upregulated in the capable group. This protein is essential for sperm–egg recognition and has been reported as a marker of sperm acrosome integrity and fertility in multiple mammalian species (Redgrove et al. 2012; Torabi et al. 2017). Its upregulation suggests enhanced sperm functional capacity in males with successful mating behaviors. Moreover, co‐expression and machine learning‐based feature selection (random forest) highlighted additional genes such as RAB3C and SOAT2. RAB3C has been implicated in regulated exocytosis and synaptic vesicle trafficking (Stenmark 2009), whereas SOAT2 is involved in cholesterol metabolism (Liang et al. 2024), a critical pathway for steroidogenesis and hormone signaling in Sèdes et al. (2018) and Shi et al. (2018). These findings suggest that mating‐capable individuals may have optimized steroid hormone biosynthesis and neural responsiveness, facilitating reproductive competence. Gene Ontology (GO) and KEGG enrichment analyses further revealed that DEGs in the capable group were enriched in signaling pathways such as reproduction, multi‐organism interaction, and hormone‐mediated processes. Notably, enrichment in the Wnt and Notch signaling pathways is consistent with their known roles in testicular development, spermatogonial stem cell maintenance, and sperm differentiation (Dong et al. 2015; Kamińska et al. 2024; Zhao, Lu, et al. 2021). These observations align with findings in other mammals where disruptions in these pathways are linked to infertility or reduced reproductive performance (Koch et al. 2015). Importantly, our study found that although PCoA analysis did not reveal pronounced transcriptomic divergence at the global level, the presence of 91 DEGs with distinct biological functions suggests that mating behavior in pandas may not be driven by broad transcriptomic remodeling but rather by fine‐tuned regulatory differences in key reproductive genes. This highlights the sensitivity of reproductive phenotypes to subtle molecular alterations. These transcriptomic insights align with emerging literature emphasizing the contribution of neuroendocrine and metabolic pathways to male reproductive behavior across taxa (Jean et al. 2021). In this context, our findings in giant pandas offer a rare example of how transcriptional regulation in peripheral tissues, such as blood, may reflect reproductive potential—an insight that could inform reproductive health monitoring in wildlife conservation programs.

Interestingly, we found that the largest category of mating‐associated genes in captive male giant pandas was related to GnRH, a neuropeptide that activates the hypothalamic–pituitary–gonadal axis to promote testosterone secretion, spermatogenesis, and testicular development (Adams 2005; Spergel 2019). Previous research has associated GnRHR polymorphisms with reproduction in female pandas (Tang et al. 2019), and our findings extend the significance of GnRH signaling to male fertility. The second‐largest gene category involved olfactory receptors (ORs), which regulate mating behaviors in insects, birds, and mammals through chemosensory and axon guidance mechanisms (Smith et al. 2013) (Del Punta et al. 2002; Höglund et al. 2017; Horth 2007; Jie et al. 2019; Kwon et al. 2021; Wang et al. 2021). In giant pandas, ORs mediate crucial social and sexual communication via chemical signals, and males with poor mating abilities often show reduced olfactory responsiveness to estrous females (Liu et al. 1998, 2008, 2006; Peng et al. 2007; Zhang et al. 2004). Furthermore, we found that functional network analysis identified key signaling modules within the MAPK pathways, which are essential for sperm maturation, testicular immune response, and maintenance of spermatogonial stem cells (Hasegawa et al. 2013; Ishii et al. 2012; Miyazaki et al. 2023; Xu et al. 2014; Zhang, Li, et al. 2021). Together, these transcriptomic patterns indicate that neuroendocrine, sensory, and intracellular signaling pathways synergistically regulate male reproductive performance in this endangered species.

In this study, we identified a significant number of DMRs between Capable and Incapable male giant pandas, primarily in CG sequence contexts, particularly within promoter and gene body regions, consistent with the CpG‐centric methylation pattern observed in mammals (Moore et al. 2013). Functional enrichment of DMR‐associated genes revealed significant involvement in key signaling pathways such as Wnt, MAPK, GnRH, and axon guidance, suggesting that epigenetic modifications may influence reproductive behavior by modulating neuroendocrine and neuronal development processes (Gardner and Pawson 2009; Hu et al. 2024; Zhu et al. 2025). Notably, hypomethylation of promoter regions in the capable group was associated with the upregulation of several reproduction‐related genes, including MAP2K1 and GnRH signaling components, supporting the classical inverse relationship between promoter methylation and gene expression (Bird 2002; Jones 2012). These findings highlight that epigenetic regulation may modulate natural mating ability in captive male giant pandas through its effects on hormonal signaling, neural circuitry, and behavioral responsiveness. The mechanisms and drivers of epigenetic regulation require further investigation, with particular emphasis on the influence of environmental factors in future studies.

Our integrative analysis of transcriptome and methylome data uncovered several key regulatory pathways associated with natural mating ability in captive male giant pandas. Notably, promoter methylation showed a more pronounced inverse correlation with gene expression than gene body methylation, reinforcing the classical model, wherein DNA methylation at promoters represses transcription (Jones 2012; Moore et al. 2013). A subset of 92 genes displayed significant inverse relationships between methylation and expression levels, suggesting these genes may be epigenetically modulated and functionally relevant to reproductive behavior. Functional enrichment of these intersecting genes highlighted their involvement in pathways related to neural regulation, immune signaling, and metabolic processes, including axon guidance, chemokine signaling, apoptosis/autophagy, and O‐glycan biosynthesis, which have been implicated in male fertility, behavioral responsiveness, and neuroimmune interactions (Hsu et al. 2024; Schagdarsurengin and Steger 2016). In particular, the significantly enriched cysteine and methionine metabolism pathway has been linked to semen quality (e.g., sperm density and motility) and antioxidant capacity, as well as elevated serum hormone levels (Zhao et al. 2025), and has also been reported to correlate with estrous peak periods in giant (Wang et al. 2024). Additionally, the observed differential CG methylation density on the X chromosome may reflect epigenetic regulation of sex‐linked genes impacting mating drive and reproductive competence, a phenomenon also documented in other mammals (Schurz et al. 2019). Together, these findings highlight a set of epigenetically modulated molecular networks that may underpin natural mating ability and offer new mechanistic insights into reproductive phenotypes in endangered species. Extending this integrative framework to other endangered species may offer novel insights into how epigenetic mechanisms shape animal behavior and reproductive fitness, with implications for conservation and captive management strategies (Zhang, Li, et al. 2021; Zhang, Zhang, et al. 2021).

## 5 Conclusion

This study integrates transcriptomic and DNA methylation data to uncover key molecular features underlying natural mating ability in captive male giant pandas. Transcriptomic analysis identified significant upregulation of genes involved in the GnRH signaling pathway, olfactory receptors, and MAPK family members in capable individuals. Notable key genes include *ZPBP2*, *PIWIL4*, and *RAF1*, which play critical roles in reproductive hormone regulation, sperm recognition and maturation, and cellular signaling, respectively. Methylation profiling revealed that promoter CG methylation changes are inversely correlated with gene expression and enriched in neural signaling, immune response, and metabolic pathways. Integrated analysis highlights coordinated epigenetic and transcriptional regulation of several key genes, emphasizing the pivotal role of DNA methylation in regulating mating ability phenotypes. These findings provide novel molecular insights into reproductive dysfunction in endangered species and offer theoretical support for captive breeding management and conservation strategies.

## Author Contributions

**Zheng Yan:** conceptualization (lead), data curation (lead), methodology (lead), software (lead), visualization (lead), writing – original draft (lead), writing – review and editing (lead). **Yinghu Lei:** conceptualization (equal). **Pengpeng Zhao:** conceptualization (equal). **Danhui Zhang:** conceptualization (equal). **Jiena Shen:** conceptualization (equal). **Guiquan Zhang:** conceptualization (equal). **Rongping Wei:** conceptualization (equal). **Mingyue Zhang:** conceptualization (equal), data curation (equal), funding acquisition (equal), resources (equal), writing – review and editing (equal). **Dingzhen Liu:** conceptualization (equal), data curation (equal), funding acquisition (equal), project administration (equal), resources (equal), writing – review and editing (equal).

## Conflicts of Interest

The authors declare no conflicts of interest.

## Supporting information


**Figure S1.** Group‐wise differential analysis of giant panda samples from two breeding facilities: CRBGPB (Sichuan) and RCQGP (Qinling). (A) and (B) show the principal coordinate analysis (PCoA) on the basis of Bray–Curtis dissimilarity for CRBGPB and RCQGP samples, respectively. Each point represents an individual sample, with color indicating group identity: Capable (red) and Incapable (blue). C and D present heatmaps of pairwise sample correlations within CRBGPB and RCQGP, respectively. Both axes denote samples, and the color scale represents the squared correlation coefficient (*R* ^2^), where values approaching 1 indicate stronger correlations. Correlations with *R* ^2^ > 0.8 are considered highly correlated.
**Figure S2.** The Gene Ontology enrichment analysis of male giant pandas was conducted on mating‐related genes. The analysis identified the top 10 significant terms in biological process (BP), cellular component (CC), and molecular function (MF).
**Figure S3.** Top 20 enriched KEGG pathways on the basis of mating‐related gene enrichment. Gene Ratio represents the ratio of differentially annotated genes to the total number of differentially expressed genes in each KEGG pathway. The size of the circles represents the number of genes.
**Figure S4.** Protein–protein interaction network of mating‐related genes. Only experimentally validated interactions are shown, with a minimum required interaction score of medium confidence 0.4. The thickness of the connecting lines indicates the magnitude of the combined score, with thicker lines representing higher scores. The darkness of the nodes represents the magnitude of betweenness centrality (BC), whereas the size of the circles represents the degree centrality.
**Figure S5.** Correlation analysis of methylation levels among samples across different sequence contexts. (A–C) Show the squared Pearson correlation coefficients (*R* ^2^) among samples for CG, CHG, and CHH methylation contexts, respectively. Higher *R* ^2^ values (> 0.8) indicate a high level of consistency in methylation patterns between samples, whereas lower *R* ^2^ values reflect greater inter‐sample variability.
**Figure S6.** Circos plot comparing CG methylation levels between Capable and Incapable groups. From outer to inner rings, the plot displays: CG methylation levels in the Capable group, methylation level differences between groups, and CG methylation levels in the Incapable group. The colored track represents regional DNA methylation levels, whereas the central heatmap illustrates methylation differences between the two groups. Color intensity indicates the magnitude of methylation or differential methylation across genomic regions.
**Figure S7.** Circos plot comparing CHG methylation levels between Capable and Incapable groups. From outer to inner rings, the plot displays: CHG methylation levels in the Capable group, methylation level differences between groups, and CHG methylation levels in the Incapable group. The colored track represents regional DNA methylation levels, whereas the central heatmap illustrates methylation differences between the two groups. Color intensity indicates the magnitude of methylation or differential methylation across genomic regions.
**Figure S8.** Circos plot comparing CHH methylation levels between Capable and Incapable groups. From outer to inner rings, the plot displays: CHH methylation levels in the Capable group, methylation level differences between groups, and CHH methylation levels in the Incapable group. The colored track represents regional DNA methylation levels, whereas the central heatmap illustrates methylation differences between the two groups. Color intensity indicates the magnitude of methylation or differential methylation across genomic regions.
**Figure S9.** Clustering heatmaps of gene body‐associated genes anchored by differentially methylated regions (DMRs) under different sequence contexts. (A) CHG sequence context; (B) CHH sequence context. The *x*‐axis represents the comparison groups (Capable vs. Incapable), and the *y*‐axis represents genes. Color gradients from blue to red indicate increasing levels of DNA methylation.
**Figure S10.** KEGG pathway enrichment analysis of DMR‐associated genes under the CG sequence context.

**Tables S1–S17:** ece372283‐sup‐0002‐TablesS1‐S2.xlsx.

## Data Availability

The transcriptome sequencing data of seven giant pandas from RCQGP generated in this study have been deposited in the Sequence Read Archive (SRA) under BioProject ID PRJNA1024053. The raw transcriptome reads for six giant pandas from CCRCGP and eight giant pandas from the CRBGPB are publicly available in the CNGB Sequence Archive (CNSA) of the China National GeneBank DataBase (CNGBdb) under accession numbers CNP0007314 and CNP0007315, respectively (Chen et al. 2020; Guo et al. 2020). Additionally, the whole‐genome bisulfite sequencing (WGBS) raw reads for eight Chengdu base giant pandas are publicly accessible in CNSA under accession number CNP0007301. The dataset containing novel giant panda gene sequences, differentially expressed genes between capable and incapable males, shared transcriptome‐methylation genes, and Supporting Information is available at the following link: https://zenodo.org/records/15807691.

## References

[ece372283-bib-0001] Adams, T. E. 2005. “Using Gonadotropin‐Releasing Hormone (GnRH) and GnRH Analogs to Modulate Testis Function and Enhance the Productivity of Domestic Animals.” Animal Reproduction Science 88: 127–139. 10.1016/j.anireprosci.2005.05.006. 15970407

[ece372283-bib-0002] Anslan, S. , V. Mikryukov , K. Armolaitis , et al. 2021. “Highly Comparable Metabarcoding Results From MGI‐Tech and Illumina Sequencing Platforms.” PeerJ 9: e12254. 10.7717/peerj.12254. 34703674 PMC8491618

[ece372283-bib-0003] Berardini, T. Z. , L. Donghui , E. Huala , et al. 2010. “The Gene Ontology in 2010: Extensions and Refinements the Gene Ontology Consortium.” Nucleic Acids Research 38: 331–335. 10.1093/nar/gkp1018. PMC2808930 19920128

[ece372283-bib-0004] Bird, A. 2002. “DNA Methylation Patterns and Epigenetic Memory.” Genes & Development 16: 6–21. 10.1101/gad.947102. 11782440

[ece372283-bib-0005] Breton‐Larrivée, M. , E. Elder , and S. McGraw . 2019. “DNA Methylation, Environmental Exposures and Early Embryo Development.” Animal Reproduction 16: 465–474. 10.21451/1984-3143-ar2019-0062. 32435290 PMC7234019

[ece372283-bib-0006] Cao, J. , and S. Zhang . 2014. “A Bayesian Extension of the Hypergeometric Test for Functional Enrichment Analysis.” Biometrics 70: 84–94. 10.1111/biom.12122. 24320951 PMC3954234

[ece372283-bib-0007] Chen, F. Z. , L. J. You , F. Yang , et al. 2020. “CNGBdb: China National GeneBank DataBase.” Yi Chuan 42: 799–809. 10.16288/j.yczz.20-080. 32952115

[ece372283-bib-0008] Chen, Y. , Y. Zheng , Y. Gao , et al. 2018. “Single‐Cell RNA‐Seq Uncovers Dynamic Processes and Critical Regulators in Mouse Spermatogenesis.” Cell Research 28: 879–896. 10.1038/s41422-018-0074-y. 30061742 PMC6123400

[ece372283-bib-0009] Clubb, R. , and G. J. Mason . 2007. “Natural Behavioural Biology as a Risk Factor in Carnivore Welfare: How Analysing Species Differences Could Help Zoos Improve Enclosures.” Applied Animal Behaviour Science 102: 303–328. 10.1016/j.applanim.2006.05.033.

[ece372283-bib-0010] Dai, Q.‐L. , J. W. Li , Y. Yang , et al. 2020. “Genetic Diversity and Prediction Analysis of Small Isolated Giant Panda Populations After Release of Individuals.” Evolutionary Bioinformatics 16: 1–9. 10.1177/1176934320939945. PMC7357131 32699496

[ece372283-bib-0011] Del Punta, K. , T. Leinders‐Zufall , I. Rodriguez , et al. 2002. “Deficient Pheromone Responses in Mice Lacking a Cluster of Vomeronasal Receptor Genes.” Nature 419: 70–74. 10.1038/nature00955. 12214233

[ece372283-bib-0012] Dong, W.‐L. , F. Q. Tan , and W. X. Yang . 2015. “Wnt Signaling in Testis Development: Unnecessary or Essential?” Gene 565: 155–165. 10.1016/j.gene.2015.04.066. 25921962

[ece372283-bib-0013] Dulac, C. , and A. T. Torello . 2003. “Molecular Detection of Pheromone Signals in Mammals: From Genes to Behaviour.” Nature Reviews Neuroscience 4: 551–562. 10.1038/nrn1140. 12838330

[ece372283-bib-0014] Feng, H. , K. N. Conneely , and H. Wu . 2014. “A Bayesian Hierarchical Model to Detect Differentially Methylated Loci From Single Nucleotide Resolution Sequencing Data.” Nucleic Acids Research 42: e69. 10.1093/nar/gku154. 24561809 PMC4005660

[ece372283-bib-0015] Gao, S. , D. Zou , L. Mao , et al. 2015. “SMAP: A Streamlined Methylation Analysis Pipeline for Bisulfite Sequencing.” GigaScience 4: 29. 10.1186/s13742-015-0070-9. 26140213 PMC4488126

[ece372283-bib-0016] Gardner, S. , and A. J. Pawson . 2009. “Emerging Targets of the GnRH Receptor: Novel Interactions With Wnt Signalling Mediators.” Neuroendocrinology 89: 241–251. 10.1159/000165377. 18946197

[ece372283-bib-0017] Green, C. D. , Q. Ma , G. L. Manske , et al. 2018. “A Comprehensive Roadmap of Murine Spermatogenesis Defined by Single‐Cell RNA‐Seq.” Developmental Cell 46: 651–667.e610. 10.1016/j.devcel.2018.07.025. 30146481 PMC6713459

[ece372283-bib-0018] Guo, X. , F. Chen , F. Gao , et al. 2020. “CNSA: A Data Repository for Archiving Omics Data.” Database: The Journal of Biological Databases and Curation 2020: baaa005. 10.1093/database/baaa055. 32705130 PMC7377928

[ece372283-bib-0019] Han, F. , Y. Dong , W. Liu , et al. 2014. “Epigenetic Regulation of sox30 Is Associated With Testis Development in Mice.” PLoS One 9: e97203. 10.1371/journal.pone.0097203. 24810894 PMC4014610

[ece372283-bib-0020] Hasegawa, K. , S. H. Namekawa , and Y. Saga . 2013. “MEK/ERK Signaling Directly and Indirectly Contributes to the Cyclical Self‐Renewal of Spermatogonial Stem Cells.” Stem Cells 31: 2517–2527. 10.1002/stem.1486. 23897718 PMC3834200

[ece372283-bib-0021] Höglund, J. , B. Wang , S. A. Sæther , et al. 2017. “Blood Transcriptomes and de Novo Identification of Candidate Loci for Mating Success in Lekking Great Snipe ( *Gallinago media* ).” Molecular Ecology 26: 3458–3471. 10.1111/mec.14118. 28345264

[ece372283-bib-0022] Holman, L. , H. Helanterä , K. Trontti , and A. S. Mikheyev . 2019. “Comparative Transcriptomics of Social Insect Queen Pheromones.” Nature Communications 10: 1593. 10.1038/s41467-019-09567-2. PMC6453924 30962449

[ece372283-bib-0023] Holt, W. V. , and A. R. Pickard . 1999. “Role of Reproductive Technologies and Genetic Resource Banks in Animal Conservation.” Reviews of Reproduction 4: 143–150. 10.1530/ror.0.0040143. 10521151

[ece372283-bib-0024] Horth, L. 2007. “Sensory Genes and Mate Choice: Evidence That Duplications, Mutations, and Adaptive Evolution Alter Variation in Mating Cue Genes and Their Receptors.” Genomics 90: 159–175. 10.1016/j.ygeno.2007.03.021. 17544617

[ece372283-bib-0025] Hou, G.‐M. , Y.‐H. Zhang , and J.‐X. Zhang . 2022. “Inheritance of Social Dominance Is Associated With Global Sperm DNA Methylation in Inbred Male Mice.” Current Zoology 69: 143–155. 10.1093/cz/zoac030. 37092005 PMC10120999

[ece372283-bib-0026] Hsu, C.‐Y. , S. A. Jasim , H. Pallathadka , et al. 2024. “A Comprehensive Insight Into the Contribution of Epigenetics in Male Infertility; Focusing on Immunological Modifications.” Journal of Reproductive Immunology 164: 104274. 10.1016/j.jri.2024.104274. 38865894

[ece372283-bib-0027] Hu, L. , X. Wang , S. Guo , et al. 2024. “Whole‐Transcriptome Sequencing Analysis to Identify Key circRNAs, miRNAs, and mRNAs in the Development of Yak Testes.” BMC Genomics 25: 824. 10.1186/s12864-024-10716-1. 39223454 PMC11367991

[ece372283-bib-0028] Ishii, K. , M. Kanatsu‐Shinohara , S. Toyokuni , and T. Shinohara . 2012. “FGF2 Mediates Mouse Spermatogonial Stem Cell Self‐Renewal via Upregulation of Etv5 and Bcl6b Through MAP2K1 Activation.” Development 139: 1734–1743. 10.1242/dev.076539. 22491947

[ece372283-bib-0029] Itai, Y. , N. Rappoport , and R. Shamir . 2023. “Integration of Gene Expression and DNA Methylation Data Across Different Experiments.” Nucleic Acids Research 51: 7762–7776. 10.1093/nar/gkad566. 37395437 PMC10450176

[ece372283-bib-0030] Jean, A. , S. Mhaouty‐Kodja , and H. Hardin‐Pouzet . 2021. “Hypothalamic Cellular and Molecular Plasticity Linked to Sexual Experience in Male Rats and Mice.” Frontiers in Neuroendocrinology 63: 100949. 10.1016/j.yfrne.2021.100949. 34687674

[ece372283-bib-0031] Jie, H. , Z. X. Xu , Y. Su , et al. 2019. “The Transcriptome Analysis of Males Musk Gland in *Moschus berezovskii* (Artiodactyla: Moschidae).” European Zoological Journal 86: 402–412. 10.1080/24750263.2019.1681525.

[ece372283-bib-0032] Jones, P. A. 2012. “Functions of DNA Methylation: Islands, Start Sites, Gene Bodies and Beyond.” Nature Reviews Genetics 13: 484–492. 10.1038/nrg3230. 22641018

[ece372283-bib-0033] Kamińska, A. , S. Lustofin , M. Brzoskwinia , et al. 2024. “Androgens and Notch Signaling Cooperate in Seminiferous Epithelium to Regulate Genes Related to Germ Cell Development and Apoptosis.” Reproductive Biology 24: 100878. 10.1016/j.repbio.2024.100878. 38490111

[ece372283-bib-0034] Kanehisa, M. , Y. Sato , M. Kawashima , M. Furumichi , and M. Tanabe . 2016. “KEGG as a Reference Resource for Gene and Protein Annotation.” Nucleic Acids Research 44: 457–462. 10.1093/nar/gkv1070. PMC4702792 26476454

[ece372283-bib-0035] Koch, S. , S. P. Acebron , J. Herbst , G. Hatiboglu , and C. Niehrs . 2015. “Post‐Transcriptional Wnt Signaling Governs Epididymal Sperm Maturation.” Cell 163: 1225–1236. 10.1016/j.cell.2015.10.029. 26590424

[ece372283-bib-0036] Krueger, F. , and S. R. Andrews . 2011. “Bismark: a Flexible Aligner and Methylation Caller for Bisulfite‐Seq Applications.” Bioinformatics 27: 1571–1572. 10.1093/bioinformatics/btr167. 21493656 PMC3102221

[ece372283-bib-0037] Krzywinski, M. , J. Schein , İ. Birol , et al. 2009. “Circos: An Information Aesthetic for Comparative Genomics.” Genome Research 19: 1639–1645. 10.1101/gr.092759.109. 19541911 PMC2752132

[ece372283-bib-0038] Kulis, M. , S. Heath , M. Bibikova , et al. 2012. “Epigenomic Analysis Detects Widespread Gene‐Body DNA Hypomethylation in Chronic Lymphocytic Leukemia.” Nature Genetics 44: 1236–1242. 10.1038/ng.2443. 23064414

[ece372283-bib-0039] Kwon, J. T. , C. Ryu , H. Lee , et al. 2021. “An Amygdala Circuit That Suppresses Social Engagement.” Nature 593: 114–118. 10.1038/s41586-021-03413-6. 33790466 PMC9251649

[ece372283-bib-0040] Langmead, B. , and S. L. Salzberg . 2012. “Fast Gapped‐Read Alignment With Bowtie 2.” Nature Methods 9: 357–359. 10.1038/nmeth.1923. 22388286 PMC3322381

[ece372283-bib-0041] Li, D. , N. J. P. Wintle , G. Zhang , et al. 2017. “Analyzing the Past to Understand the Future: Natural Mating Yields Better Reproductive Rates Than Artificial Insemination in the Giant Panda.” Biological Conservation 216: 10–17. 10.1016/j.biocon.2017.09.025.

[ece372283-bib-0042] Li, M. F. , R. R. Swaisgood , M. A. Owen , et al. 2022. “Consequences of Nescient Mating: Artificial Insemination Increases Cub Rejection in the Giant Panda ( *Ailuropoda melanoleuca* ).” Applied Animal Behaviour Science 247: 105565. 10.1016/j.applanim.2022.105565.

[ece372283-bib-0043] Liang, J. , W. Shao , P. Ni , et al. 2024. “siRNA/CS‐PLGA Nanoparticle System Targeting Knockdown Intestinal SOAT2 Reduced Intestinal Lipid Uptake and Alleviated Obesity.” Advanced Science 11: e2403442. 10.1002/advs.202403442. 39297413 PMC11516059

[ece372283-bib-0044] Liu, D. , J. M. Fang , R. Y. Sun , et al. 1998. “Behavioral Comparison in Individuals of Different Sexual Ability in Giant Panda ( *Ailuropoda melanoleuca* ).” Acta Zoologica Sinica 44: 27–34.

[ece372283-bib-0045] Liu, D. , R. P. Wei , G. Q. Zhang , et al. 2008. “Male Panda ( *Ailuropoda melanoleuca* ) Urine Contains Kinship Information.” Chinese Science Bulletin 53: 2793–2800. 10.1007/s11434-008-0373-7.

[ece372283-bib-0046] Liu, D. , H. Yuan , H. Tian , et al. 2006. “Do Anogenital Gland Secretions of Giant Panda Code for Their Sexual Ability?” Chinese Science Bulletin 51: 1986–1995. 10.1007/s11434-006-2088-y.

[ece372283-bib-0047] Love, M. I. , W. Huber , and S. Anders . 2014. “Moderated Estimation of Fold Change and Dispersion for RNA‐Seq Data With DESeq2.” Genome Biology 15: 550. 10.1186/s13059-014-0550-8. 25516281 PMC4302049

[ece372283-bib-0048] Luján, S. , E. Caroppo , C. Niederberger , et al. 2019. “Sperm DNA Methylation Epimutation Biomarkers for Male Infertility and FSH Therapeutic Responsiveness.” Scientific Reports 9: 16786. 10.1038/s41598-019-52903-1. 31727924 PMC6856367

[ece372283-bib-0049] Luo, D. D. , D. Luo , Z. He , C. Yu , and Q. Guan . 2022. “Role of p38 MAPK Signalling in Testis Development and Male Fertility.” Oxidative Medicine and Cellular Longevity 2022: 6891897. 10.1155/2022/6891897. 36092154 PMC9453003

[ece372283-bib-0050] Mao, X. Z. , X. Mao , T. Cai , J. G. Olyarchuk , and L. Wei . 2005. “Automated Genome Annotation and Pathway Identification Using the KEGG Orthology (KO) as a Controlled Vocabulary.” Bioinformatics 21: 3787–3793. 10.1093/bioinformatics/bti430. 15817693

[ece372283-bib-0051] Marker, L. , and S. J. O'Brien . 1989. “Captive Breeding of the Cheetah ( *Acinonyx jubatus* ) in North American Zoos (1871–1986).” Zoo Biology 8: 3–16. 10.1002/zoo.1430080103.

[ece372283-bib-0052] Martin, M. S. , M. Owen , N. J. P. Wintle , G. Zhang , H. Zhang , and R. R. Swaisgood . 2020. “Stereotypic Behaviour Predicts Reproductive Performance and Litter Sex Ratio in Giant Pandas.” Scientific Reports 10: 7263. 10.1038/s41598-020-63763-5. 32350317 PMC7190838

[ece372283-bib-0054] Martin‐Wintle, M. S. , D. Shepherdson , G. Zhang , et al. 2015. “Free Mate Choice Enhances Conservation Breeding in the Endangered Giant Panda.” Nature Communications 6: 10125. 10.1038/ncomms10125. PMC4682106 26670381

[ece372283-bib-0053] Martin‐Wintle, M. S. , D. Shepherdson , G. Zhang , Y. Huang , B. Luo , and R. R. Swaisgood . 2017. “Do Opposites Attract? Effects of Personality Matching in Breeding Pairs of Captive Giant Pandas on Reproductive Success.” Biological Conservation 207: 27–37. 10.1016/j.biocon.2017.01.010.

[ece372283-bib-0055] Mason, G. , R. Clubb , N. Latham , and S. Vickery . 2007. “Why and How Should We Use Environmental Enrichment to Tackle Stereotypic Behaviour?” Applied Animal Behaviour Science 102: 163–188. 10.1016/j.applanim.2006.05.041.

[ece372283-bib-0056] McCabe, C. F. , V. Padmanabhan , D. C. Dolinoy , et al. 2020. “Maternal Environmental Exposure to Bisphenols and Epigenome‐Wide DNA Methylation in Infant Cord Blood.” Environmental Epigenetics 6: dvaa021. 10.1093/eep/dvaa021. 33391824 PMC7757124

[ece372283-bib-0057] Mishra, D. K. , Z. Chen , Y. Wu , M. Sarkissyan , H. P. Koeffler , and J. V. Vadgama . 2010. “Global Methylation Pattern of Genes in Androgen‐Sensitive and Androgen‐Independent Prostate Cancer Cells.” Molecular Cancer Therapeutics 9: 33–45. 10.1158/1535-7163.Mct-09-0486. 20053773 PMC2806502

[ece372283-bib-0058] Miyazaki, T. , M. Kanatsu‐Shinohara , M. Ema , and T. Shinohara . 2023. “Signal Regulatory Protein Alpha Is a Conserved Marker for Mouse and Rat Spermatogonial Stem Cells.” Biology of Reproduction 108: 682–693. 10.1093/biolre/ioad006. 36648447

[ece372283-bib-0059] Moore, L. D. , T. le , and G. Fan . 2013. “DNA Methylation and Its Basic Function.” Neuropsychopharmacology 38: 23–38. 10.1038/npp.2012.112. 22781841 PMC3521964

[ece372283-bib-0060] Peng, J. J. , J. Peng , Z. Jiang , et al. 2007. “Impact of Activity Space on the Reproductive Behaviour of Giant Panda (*Ailuropoda melanoleuca*) in Captivity.” Applied Animal Behaviour Science 104: 151–161. 10.1016/j.applanim.2006.04.029.

[ece372283-bib-0061] Pertea, M. , G. M. Pertea , C. M. Antonescu , T. C. Chang , J. T. Mendell , and S. L. Salzberg . 2015. “StringTie Enables Improved Reconstruction of a Transcriptome From RNA‐Seq Reads.” Nature Biotechnology 33: 290–295. 10.1038/nbt.3122. PMC4643835 25690850

[ece372283-bib-0062] Price, E. O. 1999. “Behavioral Development in Animals Undergoing Domestication.” Applied Animal Behaviour Science 65: 245–271. 10.1016/S0168-1591(99)00087-8.

[ece372283-bib-0063] Redgrove, K. A. , B. Nixon , M. A. Baker , et al. 2012. “The Molecular Chaperone HSPA2 Plays a Key Role in Regulating the Expression of Sperm Surface Receptors That Mediate Sperm‐Egg Recognition.” PLoS One 7: e50851. 10.1371/journal.pone.0050851. 23209833 PMC3510172

[ece372283-bib-0064] Santamarina‐Ojeda, P. , J. R. Tejedor , R. F. Pérez , et al. 2023. “Multi‐Omic Integration of DNA Methylation and Gene Expression Data Reveals Molecular Vulnerabilities in Glioblastoma.” Molecular Oncology 17: 1726–1743. 10.1002/1878-0261.13479. 37357610 PMC10483606

[ece372283-bib-0065] Schagdarsurengin, U. , and K. Steger . 2016. “Epigenetics in Male Reproduction: Effect of Paternal Diet on Sperm Quality and Offspring Health.” Nature Reviews Urology 13: 584–595. 10.1038/nrurol.2016.157. 27578043

[ece372283-bib-0066] Schurz, H. , M. Salie , G. Tromp , E. G. Hoal , C. J. Kinnear , and M. Möller . 2019. “The X Chromosome and Sex‐Specific Effects in Infectious Disease Susceptibility.” Human Genomics 13: 2. 10.1186/s40246-018-0185-z. 30621780 PMC6325731

[ece372283-bib-0067] Seddon, P. J. , C. J. Griffiths , P. S. Soorae , and D. P. Armstrong . 2014. “Reversing Defaunation: Restoring Species in a Changing World.” Science 345: 406–412. 10.1126/science.1251818. 25061203

[ece372283-bib-0068] Sèdes, L. , L. Thirouard , S. Maqdasy , et al. 2018. “Cholesterol: A Gatekeeper of Male Fertility?” Frontiers in Endocrinology (Lausanne) 9: 369. 10.3389/fendo.2018.00369. PMC6060264 30072948

[ece372283-bib-0069] Shepherdson, D. 1994. “The Role of Environmental Enrichment in the Captive Breeding and Reintroduction of Endangered Species.” In Creative Conservation: Interactive Management of Wild and Captive Animals, edited by P. J. S. Olney , G. M. Mace , and A. T. C. Feistner , 167–177. Springer Netherlands.

[ece372283-bib-0070] Shi, J. F. , Y. K. Li , K. Ren , et al. 2018. “Characterization of Cholesterol Metabolism in Sertoli Cells and Spermatogenesis.” Molecular Medicine Reports 17: 705–713. 10.3892/mmr.2017.8000. 29115523 PMC5780145

[ece372283-bib-0071] Smallwood, S. A. , H. J. Lee , C. Angermueller , et al. 2014. “Single‐Cell Genome‐Wide Bisulfite Sequencing for Assessing Epigenetic Heterogeneity.” Nature Methods 11: 817–820. 10.1038/nmeth.3035. 25042786 PMC4117646

[ece372283-bib-0072] Smith, G. , Y. Fang , X. Liu , et al. 2013. “Transcriptome‐Wide Expression Variation Associated With Environmental Plasticity and Mating Success in Cactophilic *Drosophila mojavensis* .” Evolution 67: 1950–1963. 10.1111/evo.12082. 23815652

[ece372283-bib-0073] Song, Q. , B. Decato , E. E. Hong , et al. 2013. “A Reference Methylome Database and Analysis Pipeline to Facilitate Integrative and Comparative Epigenomics.” PLoS One 8: e81148. 10.1371/journal.pone.0081148. 24324667 PMC3855694

[ece372283-bib-0074] Spergel, D. J. 2019. “Neuropeptidergic Modulation of GnRH Neuronal Activity and GnRH Secretion Controlling Reproduction: Insights From Recent Mouse Studies.” Cell and Tissue Research 375: 179–191. 10.1007/s00441-018-2893-z. 30078104

[ece372283-bib-0075] Stenmark, H. 2009. “Rab GTPases as Coordinators of Vesicle Traffic.” Nature Reviews Molecular Cell Biology 10: 513–525. 10.1038/nrm2728. 19603039

[ece372283-bib-0076] Tang, B. , X. Huang , C. Han , et al. 2019. “Snp Detection of *GNRHR* Gene and Its Association With Litter Size Traits in Giant Panda.” Journal of Animal and Plant Sciences 29: 461–466.

[ece372283-bib-0077] Tipkantha, W. , P. Thuwanut , U. Maikeaw , et al. 2017. “Successful Laparoscopic Oviductal Artificial Insemination in the Clouded Leopard ( *Neofelis nebulosa* ) in Thailand.” Journal of Zoo and Wildlife Medicine 48: 804–812. 28920796 10.1638/2016-0287.1

[ece372283-bib-0078] Torabi, F. , O. A. Bogle , J. M. Estanyol , R. Oliva , and D. Miller . 2017. “Zona Pellucida‐Binding Protein 2 (ZPBP2) and Several Proteins Containing BX7B Motifs in Human Sperm May Have Hyaluronic Acid Binding or Recognition Properties.” Molecular Human Reproduction 23: 803–816. 10.1093/molehr/gax053. 29126140 PMC5909853

[ece372283-bib-0079] Wang, D. H. , D. Wang , J. Chen , et al. 2024. “Biochemical Characteristics of Urine Metabolomics in Female Giant Pandas at Different Estrous Stages.” Animals 14: 3486. 10.3390/ani14233486. 39682452 PMC11640436

[ece372283-bib-0080] Wang, G. , J. Vega‐Rodríguez , A. Diabate , et al. 2021. “Clock Genes and Environmental Cues Coordinate Anopheles Pheromone Synthesis, Swarming, and Mating.” Science 371: 411–415. 10.1126/science.abd4359. 33479155 PMC9854397

[ece372283-bib-0081] Wang, M. , D. Yuan , L. Tu , et al. 2015. “Long Noncoding RNAs and Their Proposed Functions in Fibre Development of Cotton (*Gossypium* spp.).” New Phytologist 207: 1181–1197. 10.1111/nph.13429. 25919642

[ece372283-bib-0082] Wei, F. , Y. Hu , L. Zhu , M. W. Bruford , X. Zhan , and L. Zhang . 2012. “Black and White and Read all Over: the Past, Present and Future of Giant Panda Genetics.” Molecular Ecology 21: 5660–5674. 10.1111/mec.12096. 23130639

[ece372283-bib-0083] Wickham, H. 2011. “ggplot2.” WIREs Computational Statistics 3: 180–185. 10.1002/wics.147.

[ece372283-bib-0084] Wildt, D. E. , P. Comizzoli , B. Pukazhenthi , and N. Songsasen . 2010. “Lessons From Biodiversity—The Value of Nontraditional Species to Advance Reproductive Science, Conservation, and Human Health.” Molecular Reproduction and Development 77: 397–409. 10.1002/mrd.21137. 19967718 PMC3929270

[ece372283-bib-0085] Wu, H. , T. Xu , H. Feng , et al. 2015. “Detection of Differentially Methylated Regions From Whole‐Genome Bisulfite Sequencing Data Without Replicates.” Nucleic Acids Research 43: gkv715. 10.1093/nar/gkv715. PMC4666378 26184873

[ece372283-bib-0086] Xu, B. , A. M. Washington , and B. T. Hinton . 2014. “PTEN Signaling Through RAF1 Proto‐Oncogene Serine/Threonine Kinase (RAF1)/ERK in the Epididymis Is Essential for Male Fertility.” Proceedings of the National Academy of Sciences of the United States of America 111: 18643–18648. 10.1073/pnas.1413186112. 25512490 PMC4284526

[ece372283-bib-0087] Xu, J. , S. Zhou , X. Gong , et al. 2018. “Single‐Base Methylome Analysis Reveals Dynamic Epigenomic Differences Associated With Water Deficit in Apple.” Plant Biotechnology Journal 16: 672–687. 10.1111/pbi.12820. 28796917 PMC5787839

[ece372283-bib-0088] Yan, Z. , Y. Lei , P. Zhao , et al. 2024. “Natural Mating Ability Is Associated With Gut Microbiota Composition and Function in Captive Male Giant Pandas.” Ecology and Evolution 14: e11189. 10.1002/ece3.11189. 38571808 PMC10985376

[ece372283-bib-0089] Yan, Z. , Y. Yao , Q. Xu , X. He , X. Zhou , and H. Wang . 2025. “Dietary Microbiota‐Mediated Shifts in Gut Microbial Ecology and Pathogen Interactions in Giant Pandas ( *Ailuropoda melanoleuca* ).” Communications Biology 8: 864. 10.1038/s42003-025-08270-x. 40467767 PMC12137640

[ece372283-bib-0090] Yang, I. V. , B. S. Pedersen , E. Rabinovich , et al. 2014. “Relationship of DNA Methylation and Gene Expression in Idiopathic Pulmonary Fibrosis.” American Journal of Respiratory and Critical Care Medicine 190: 1263–1272. 10.1164/rccm.201408-1452OC. 25333685 PMC4315819

[ece372283-bib-0091] Young, M. D. , M. J. Wakefield , G. K. Smyth , and A. Oshlack . 2010. “Gene Ontology Analysis for RNA‐Seq: Accounting for Selection Bias.” Genome Biology 11: R14. 10.1186/gb-2010-11-2-r14. 20132535 PMC2872874

[ece372283-bib-0092] Zhang, G. Q. , G. Zhang , R. R. Swaisgood , and H. Zhang . 2004. “Evaluation of Behavioral Factors Influencing Reproductive Success and Failure in Captive Giant Pandas.” Zoo Biology 23: 15–31. 10.1002/zoo.10118.

[ece372283-bib-0093] Zhang, J. , X. Li , R. Wang , et al. 2024. “DNA Methylation Patterns in Patients With Asthenospermia and Oligoasthenospermia.” BMC Genomics 25: 602. 10.1186/s12864-024-10491-z. 38886667 PMC11181631

[ece372283-bib-0094] Zhang, M. Y. , X. Y. Wang , J. Ayala , et al. 2022. “Combined Urine Metabolomics and 16S rDNA Sequencing Analyses Reveals Physiological Mechanism Underlying Decline in Natural Mating Behavior of Captive Giant Pandas.” Frontiers in Microbiology 13: 906737. 10.3389/fmicb.2022.906737. 36118243 PMC9478395

[ece372283-bib-0095] Zhang, M. Y. , X. H. Zhang , X. Y. Wang , et al. 2023. “Intestinal Acetic Acid Regulates the Synthesis of Sex Pheromones in Captive Giant Pandas.” Frontiers in Microbiology 14: 1234676. 10.3389/fmicb.2023.1234676. 37692393 PMC10485365

[ece372283-bib-0096] Zhang, M. Y. , X. H. Zhang , P. Zhang , et al. 2021. “Natural Reproductive Performance Is Associated With Captive Management in Adult Male Giant Pandas.” Applied Animal Behaviour Science 240: 105353. 10.1016/j.applanim.2021.105353.

[ece372283-bib-0097] Zhang, Y. , Y. Li , J. Zhang , et al. 2021. “Cadmium Induced Inflammation and Apoptosis of Porcine Epididymis via Activating RAF1/MEK/ERK and NF‐κB Pathways.” Toxicology and Applied Pharmacology 415: 115449. 10.1016/j.taap.2021.115449. 33577919

[ece372283-bib-0098] Zhao, D. , Y. Shi , X. Long , Q. Tan , J. Yang , and H. Li . 2025. “Effects of Dendrobium Nobile on Antioxidant Capacity, Hormone Levels, Testicular Metabolism, and Reproductive Performance of Aged Roosters.” PLoS One 20: e0322853. 10.1371/journal.pone.0322853. 40344569 PMC12064193

[ece372283-bib-0099] Zhao, J. , P. Lu , C. Wan , et al. 2021. “Cell‐Fate Transition and Determination Analysis of Mouse Male Germ Cells Throughout Development.” Nature Communications 12: 6839. 10.1038/s41467-021-27172-0. PMC8617176 34824237

[ece372283-bib-0100] Zhao, Y. , M. C. Li , M. M. Konaté , et al. 2021. “TPM, FPKM, or Normalized Counts? A Comparative Study of Quantification Measures for the Analysis of RNA‐Seq Data From the NCI Patient‐Derived Models Repository.” Journal of Translational Medicine 19: 269. 10.1186/s12967-021-02936-w. 34158060 PMC8220791

[ece372283-bib-0101] Zhu, Y. , J. Liu , J. Wang , et al. 2025. “Integrative Transcriptomic and Epigenomic Analysis Identifies BCL6B as a Novel Regulator of Human Pluripotent Stem Cell to Endothelial Differentiation.” Protein & Cell 2025: pwaf039. 10.1093/procel/pwaf039.40318187

