# Supplementary material for: Coordinated Transcriptomic and Epigenetic Approach Reveals Molecular Features Underlying Natural Mating Ability in Captive Male Giant Pandas
Source: Ecol Evol. 2025 Oct 12;15(10):e72283. doi: 10.1002/ece3.72283 (PMC12516017; doi:10.1002/ece3.72283)
Supplement: Supplementary file 1 — Figure S1. Group‐wise differential analysis of giant panda samples from two breeding facilities: CRBGPB (Sichuan) and RCQGP (Qinling). (A) and (B) show the principal coordinate analysis (PCoA) on the basis of Bray–Curtis dissimilarity for CRBGPB and RCQGP samples, respectively. Each point represents an individual sample, with color indicating group identity: Capable (red) and Incapable (blue). C and D present heatmaps of pairwise sample correlations within CRBGPB and RCQGP, respectively. Both axes denote samples, and the color scale represents the squared correlation coefficient (R 2), where values approaching 1 indicate stronger correlations. Correlations with R 2 > 0.8 are considered highly correlated. Figure S2. The Gene Ontology enrichment analysis of male giant pandas was conducted on mating‐related genes. The analysis identified the top 10 significant terms in biological process (BP), cellular component (CC), and molecular function (MF). Figure S3. Top 20 enriched KEGG pathways on the basis of mating‐related gene enrichment. Gene Ratio represents the ratio of differentially annotated genes to the total number of differentially expressed genes in each KEGG pathway. The size of the circles represents the number of genes. Figure S4. Protein–protein interaction network of mating‐related genes. Only experimentally validated interactions are shown, with a minimum required interaction score of medium confidence 0.4. The thickness of the connecting lines indicates the magnitude of the combined score, with thicker lines representing higher scores. The darkness of the nodes represents the magnitude of betweenness centrality (BC), whereas the size of the circles represents the degree centrality. Figure S5. Correlation analysis of methylation levels among samples across different sequence contexts. (A–C) Show the squared Pearson correlation coefficients (R 2) among samples for CG, CHG, and CHH methylation contexts, respectively. Higher R 2 values (> 0.8) indicate a high l [file ECE3-15-e72283-s001.docx]

# Supplementary Material

# Coordinated transcriptomic and epigenetic regulation reveals molecular features underlying natural mating ability in captive male giant pandas

Zheng Yan^1^, Yinghu Lei^3^, Pengpeng Zhao^3^, Danhui Zhang^3^, Jiena Shen^3^, Guiquan Zhang^4^, Rongping Wei^4^, Mingyue Zhang ^2*^, Dingzhen Liu^1*^

^1^Key Laboratory for Biodiversity and Ecological Engineering of Ministry of Education, Department of Ecology, College of Life Sciences, Beijing Normal University, Beijing, 100875, China.

^2^Sichuan Key Laboratory of Conservation Biology for Endangered Wildlife, Chengdu Research Base of Giant Panda Breeding, Chengdu, 610081, China.

^3^Research Center for the Qinling Giant Panda, Shaanxi Rare Wildlife Rescue Base, Xian, 710402, China.

^4^China Conservation and Research Center for the Giant Panda, Wolong, 623006, China.

Supplementary figures


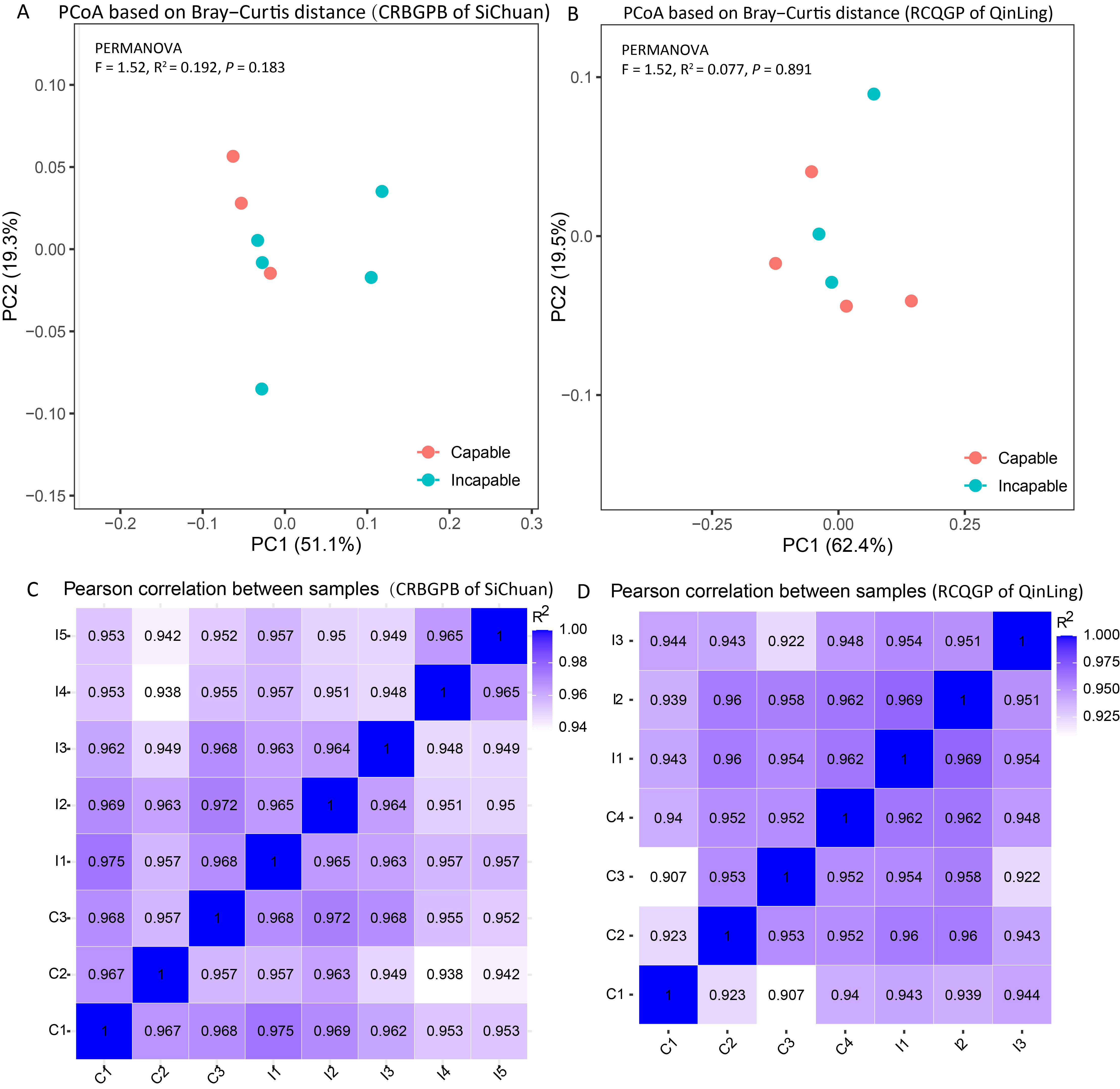


Fig. S1. Group-wise differential analysis of giant panda samples from two breeding facilities: CRBGPB (Sichuan) and RCQGP (Qinling). A and B show the principal coordinate analysis (PCoA) based on Bray–Curtis dissimilarity for CRBGPB and RCQGP samples, respectively. Each point represents an individual sample, with color indicating group identity: Capable (red) and Incapable (blue). C and D present heatmaps of pairwise sample correlations within CRBGPB and RCQGP, respectively. Both axes denote samples, and the color scale represents the squared correlation coefficient (R²), where values approaching 1 indicate stronger correlations. Correlations with R² > 0.8 are considered highly correlated.


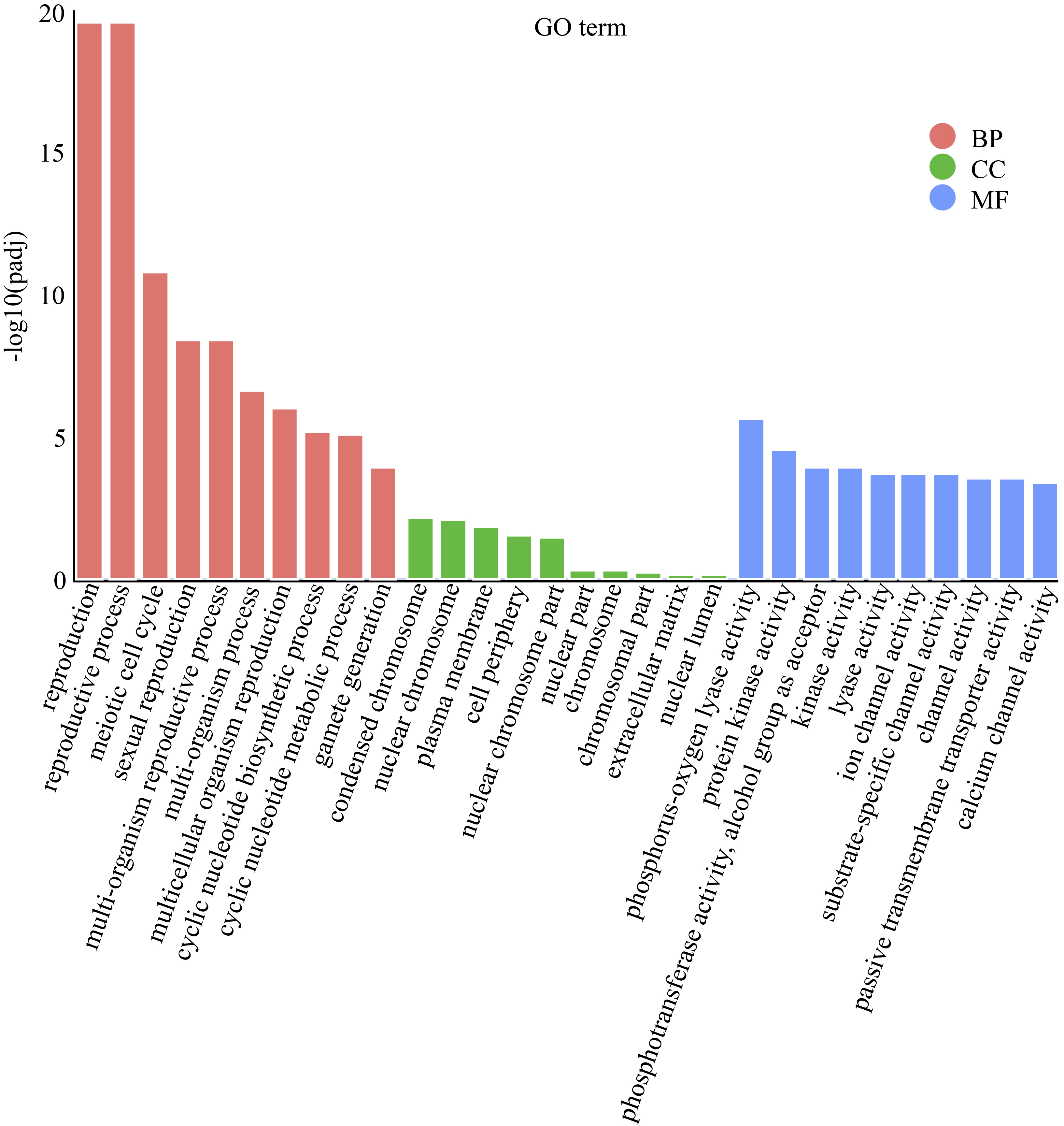


Fig. S2. The Gene Ontology enrichment analysis of male giant pandas was conducted on mating related genes. The analysis identified the top 10 significant terms in biological process (BP), cellular component (CC), and molecular function (MF).


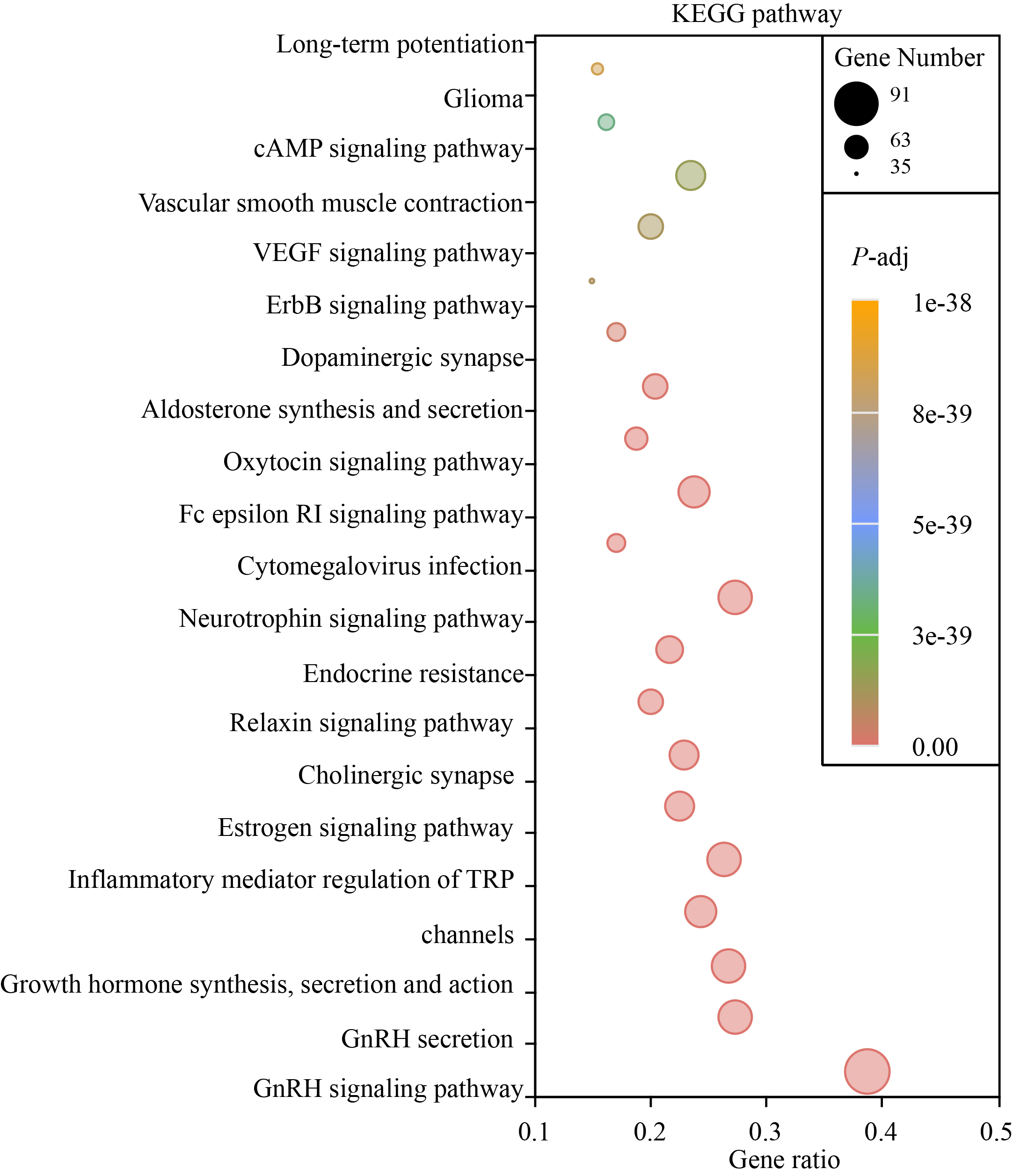


Fig. S3. Top 20 enriched KEGG pathways based on mating related genes enrichment. Gene Ratio represents the ratio of differentially annotated genes to the total number of differentially expressed genes in each KEGG pathway. The size of the circles represents the number of genes.


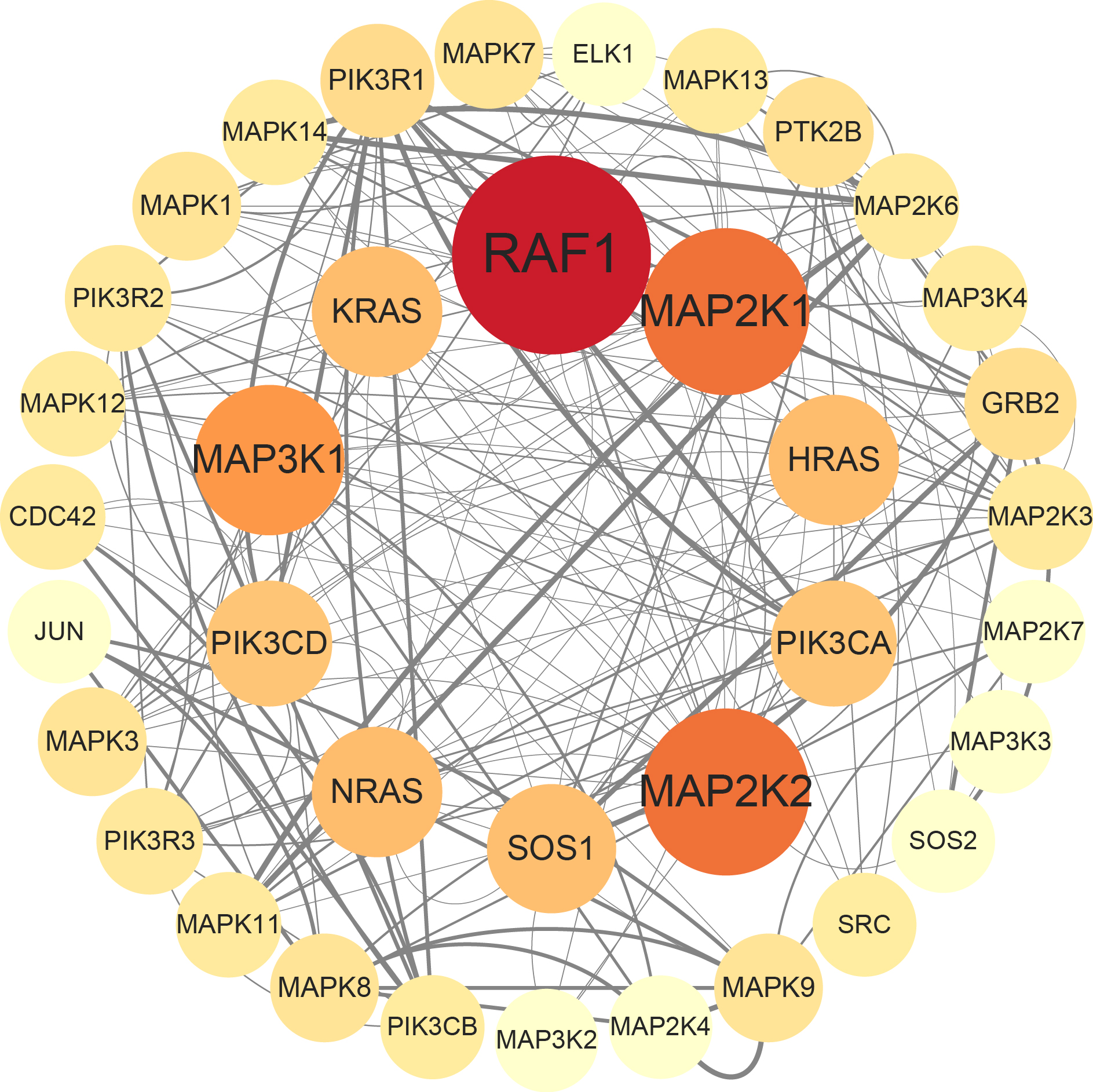


Fig. S4. Protein-protein interaction network of mating related genes. Only experimentally validated interactions are shown, with a minimum required interaction score of medium confidence 0.4. The thickness of the connecting lines indicates the magnitude of the combined score, with thicker lines representing higher scores. The darkness of the nodes represents the magnitude of betweenness centrality (BC), while the size of the circles represents the degree centrality.


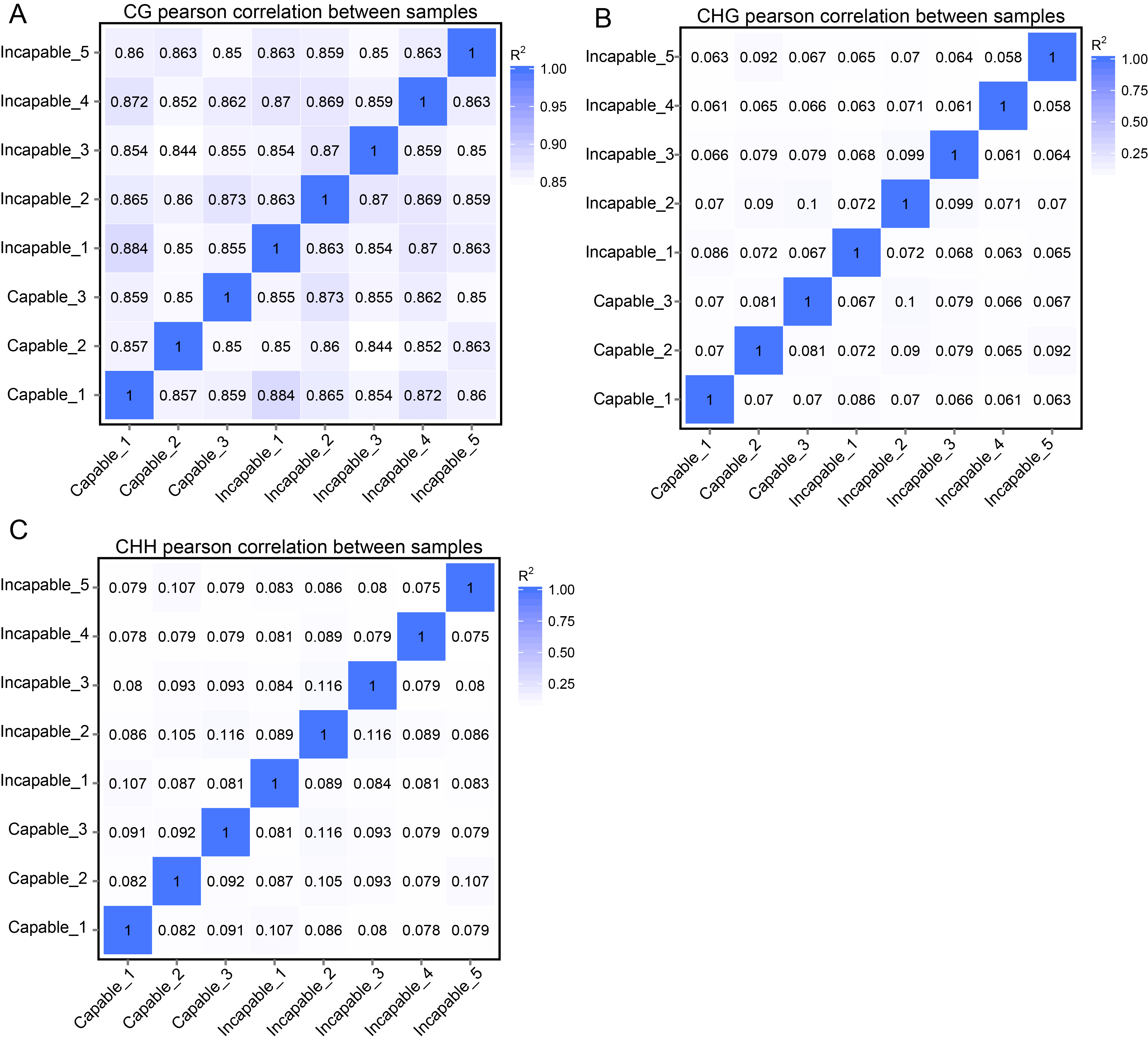


Fig. S5. Correlation analysis of methylation levels among samples across different sequence contexts. A, B, and C show the squared Pearson correlation coefficients (R²) among samples for CG, CHG, and CHH methylation contexts, respectively. Higher R² values (> 0.8) indicate a high level of consistency in methylation patterns between samples, whereas lower R² values reflect greater inter-sample variability.


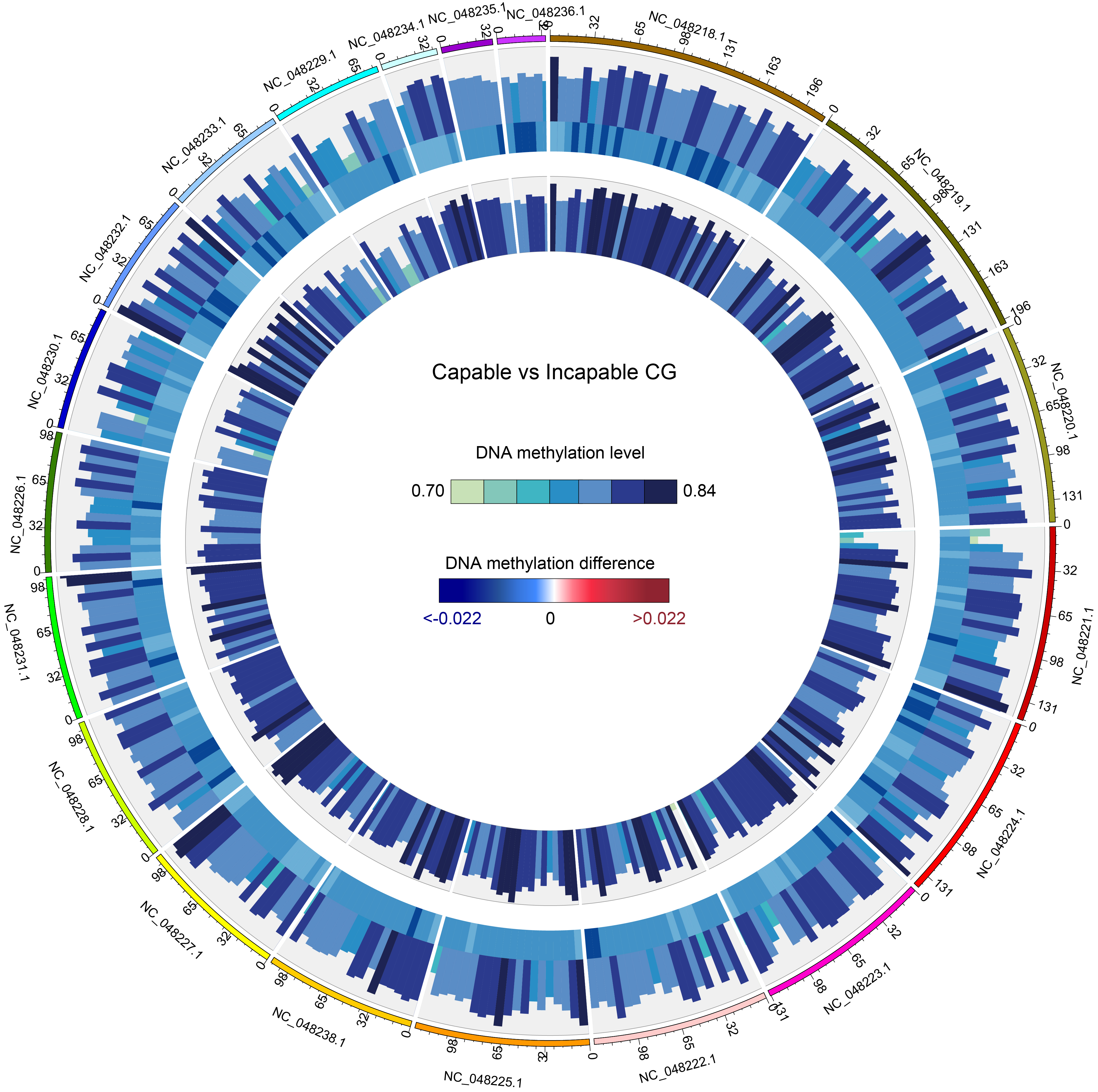


Fig. S6. Circos plot comparing CG methylation levels between Capable and Incapable groups. From outer to inner rings, the plot displays: CG methylation levels in the Capable group, methylation level differences between groups, and CG methylation levels in the Incapable group. The colored track represents regional DNA methylation levels, while the central heatmap illustrates methylation differences between the two groups. Color intensity indicates the magnitude of methylation or differential methylation across genomic regions.


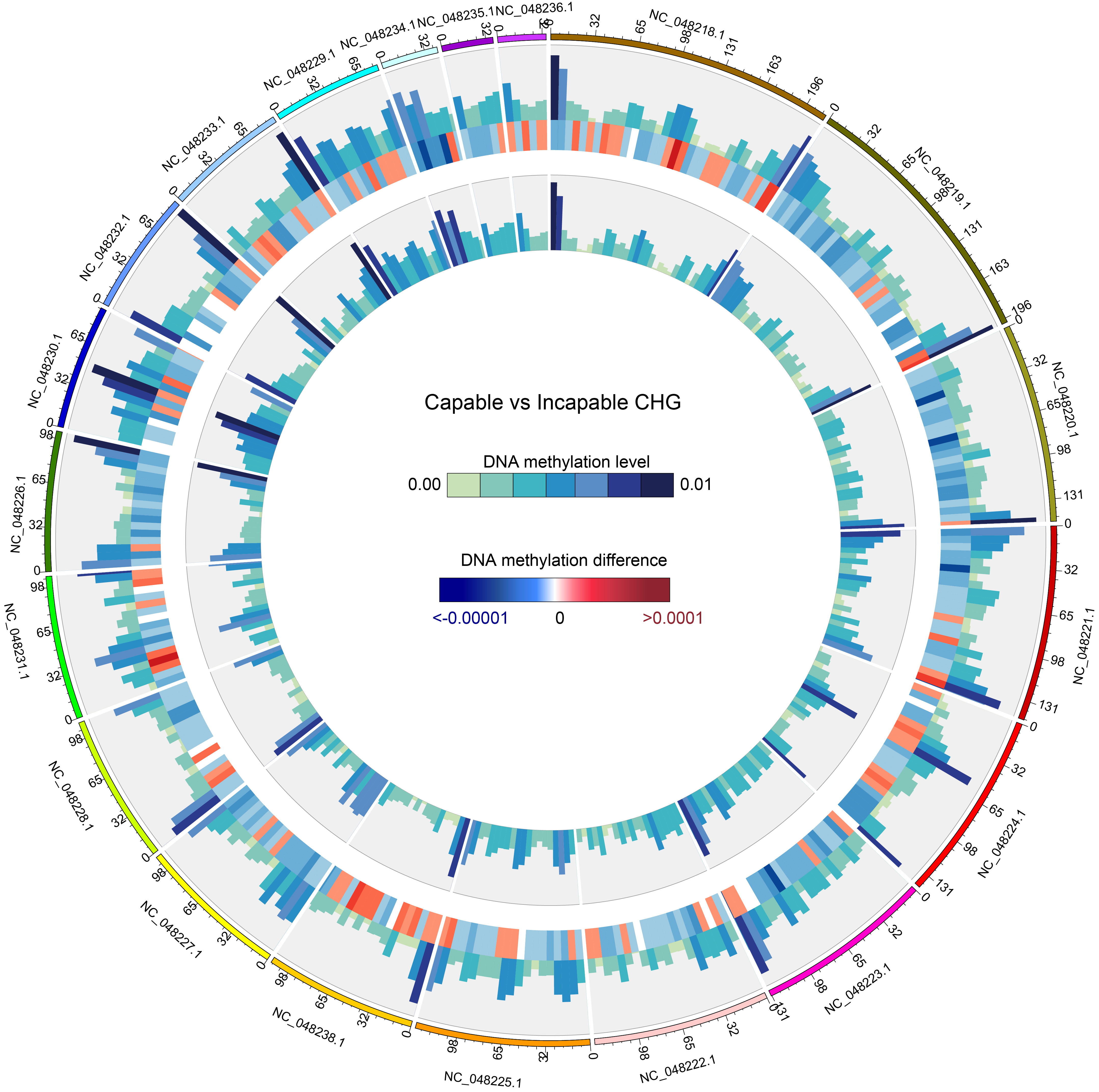


Fig. S7. Circos plot comparing CHG methylation levels between Capable and Incapable groups. From outer to inner rings, the plot displays: CHG methylation levels in the Capable group, methylation level differences between groups, and CHG methylation levels in the Incapable group. The colored track represents regional DNA methylation levels, while the central heatmap illustrates methylation differences between the two groups. Color intensity indicates the magnitude of methylation or differential methylation across genomic regions.


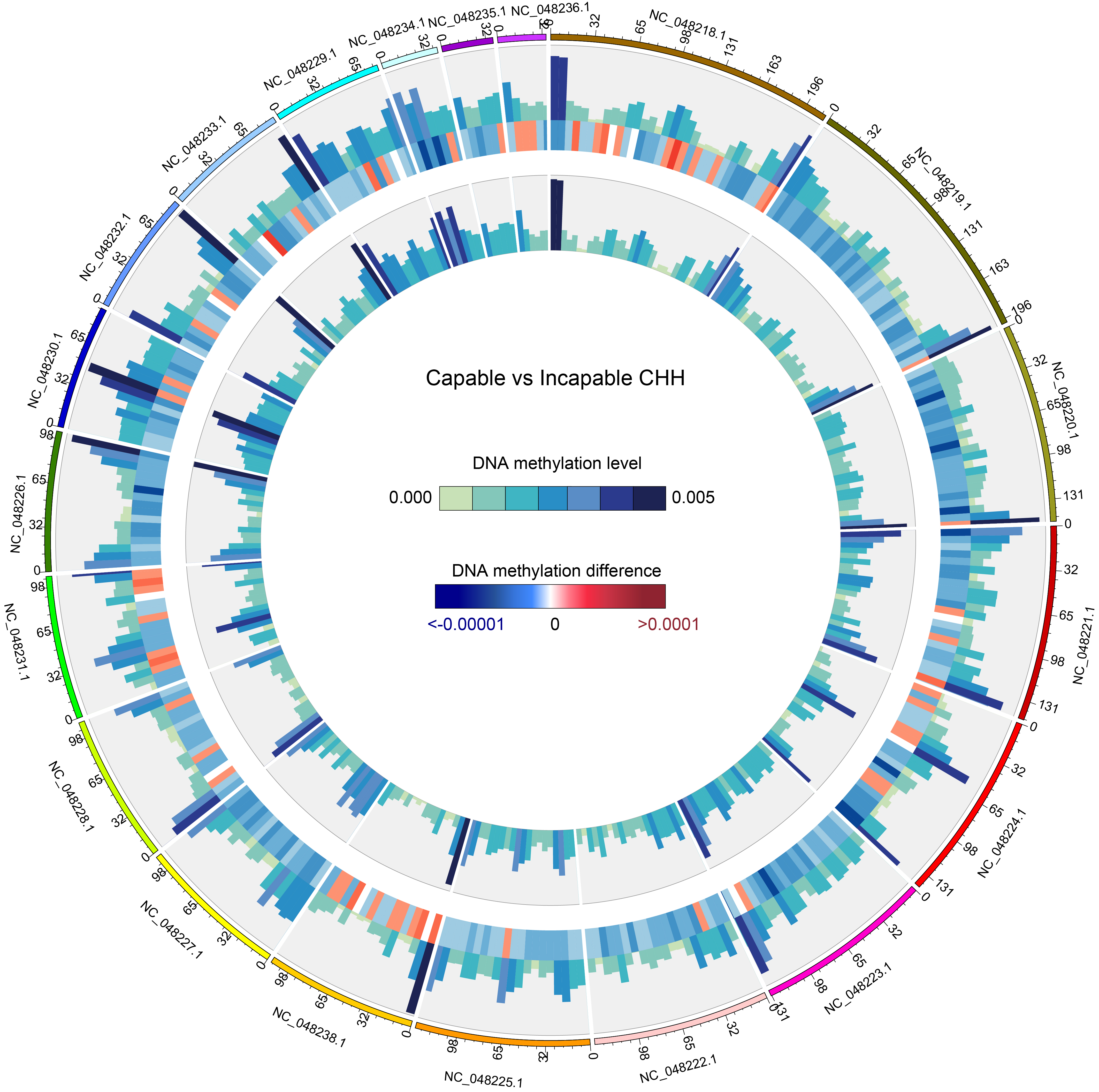


Fig. S8. Circos plot comparing CHH methylation levels between Capable and Incapable groups. From outer to inner rings, the plot displays: CHH methylation levels in the Capable group, methylation level differences between groups, and CHH methylation levels in the Incapable group. The colored track represents regional DNA methylation levels, while the central heatmap illustrates methylation differences between the two groups. Color intensity indicates the magnitude of methylation or differential methylation across genomic regions.


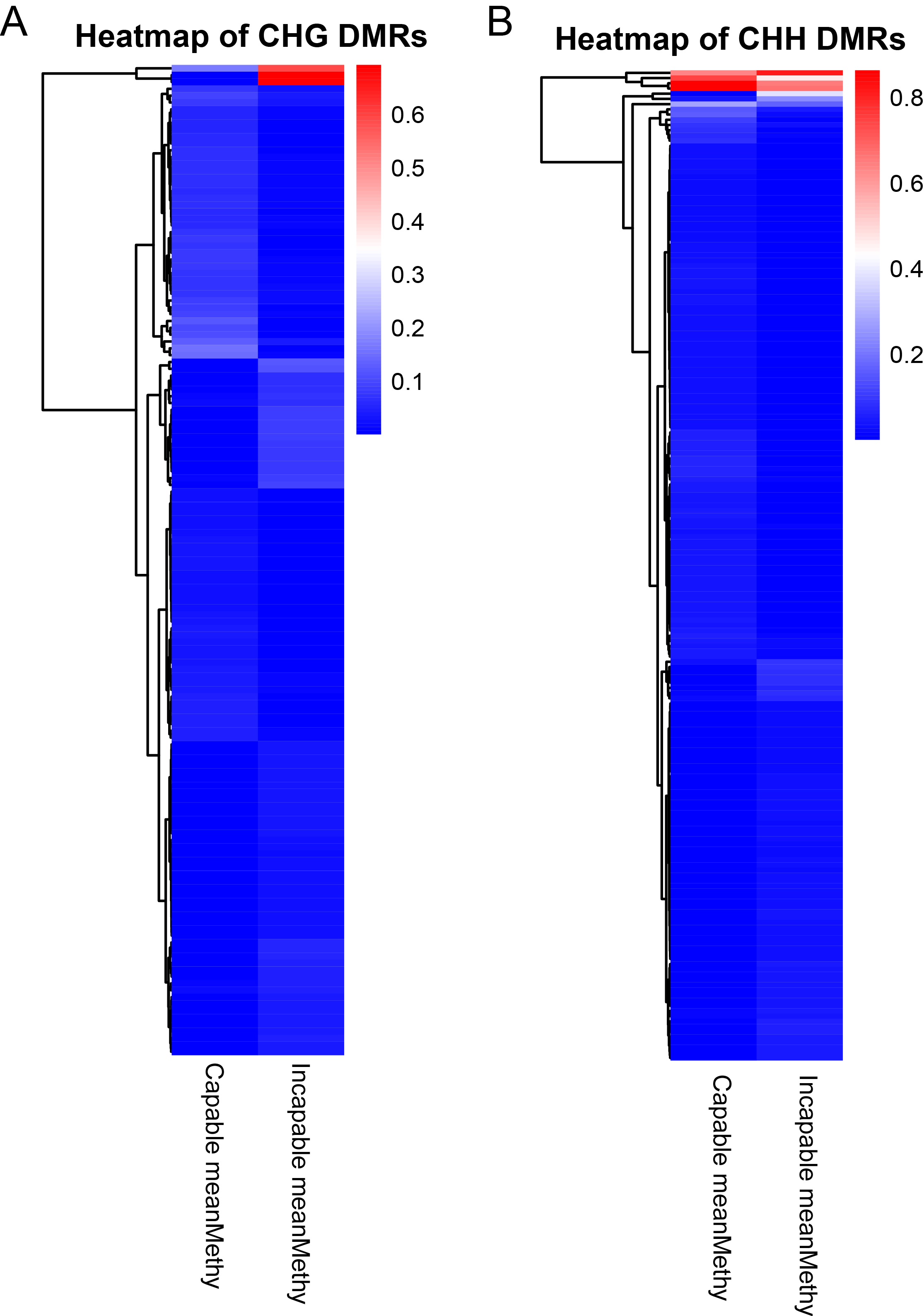


Fig. S9. Clustering heatmaps of gene body-associated genes anchored by DMRs under different sequence contexts.A: CHG sequence context; B: CHH sequence context.The x-axis represents the comparison groups (Capable vs. Incapable), and the y-axis represents genes.Color gradients from blue to red indicate increasing levels of DNA methylation.


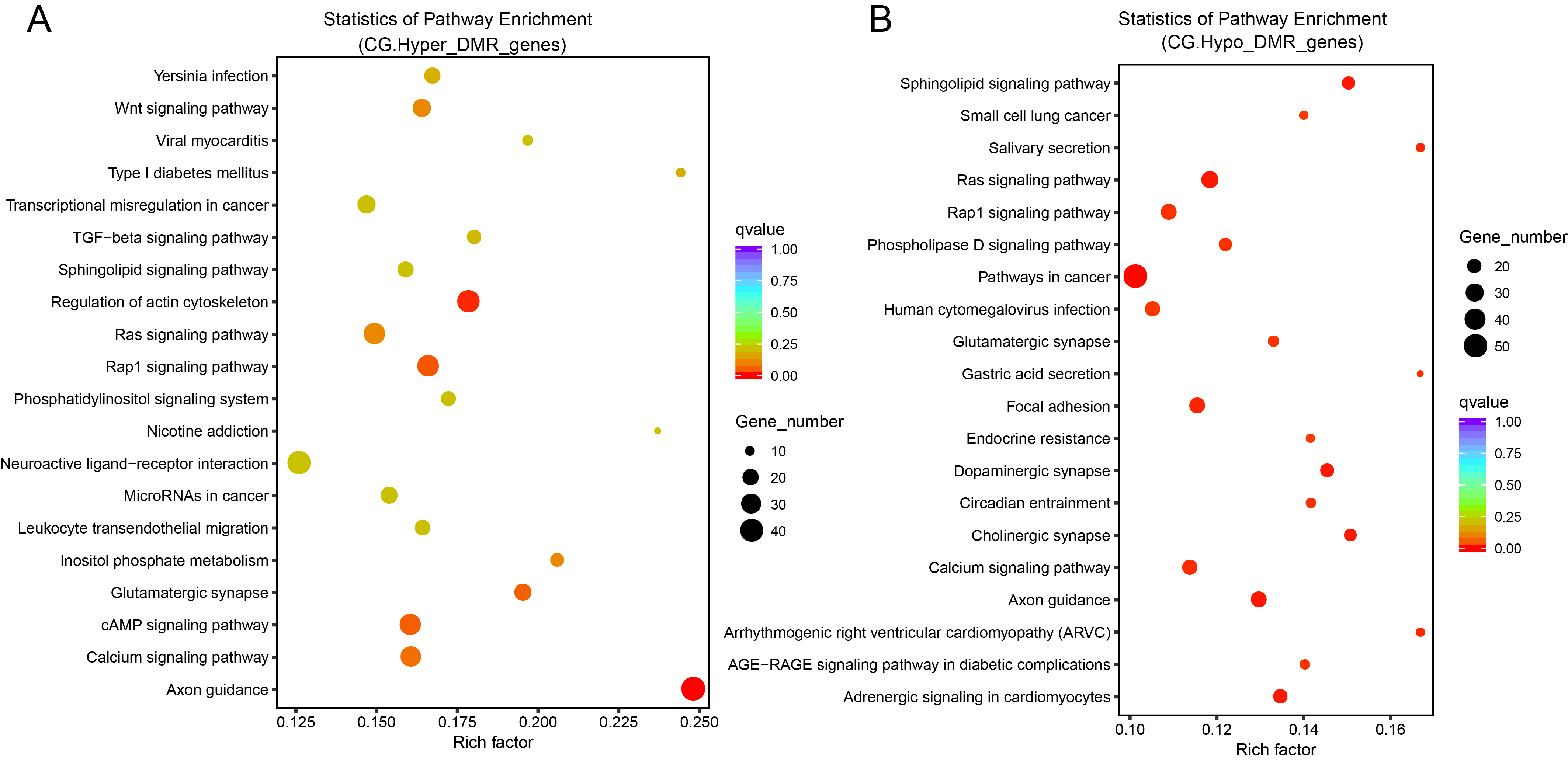


Fig. S10. KEGG pathway enrichment analysis of DMR-associated genes under the CG sequence context.

A: KEGG enrichment of hypermethylated (Hyper) DMR-associated genes in the Capable group.

B: KEGG enrichment of hypomethylated (Hypo) DMR-associated genes in the Capable group.

The y-axis represents pathway names, and the x-axis shows the Rich factor. Dot size indicates the number of DMR-associated genes involved in each pathway, while dot color reflects the corresponding Q-value range.

# Supplementary Results

## 1. Identification and analysis of genes potentially associated with sexual behavior

### 1.1 Categorization and possible functions of genes related to sexual behavior

By comparing the genes of male giant pandas with the NR databases, we extracted 320 genes directly associated with mating behavior (Table S7 and S8). Based on their functions, these genes can be divided into six categories: androgen, gonadotropin-releasing hormone (GnRH), sperm quality (spermatogenesis, motility, binding and fertilization process), olfactory receptor (OR), dopamine, and germ cell development. Furthermore, by analyzing the gene expression results (FPKM), we found that the top 20 genes with the highest expression levels among the mating related genes belonged to the GnRH category.

The Gene Ontology (GO) analysis showed that mating related genes were significantly enriched in three categories (Fig. S2). In the biological process (BP) category, terms included reproduction, meiotic cell cycle, sexual reproduction, multi-organism reproductive process, multicellular organism reproduction, cyclic nucleotide biosynthetic/metabolic process, and gamete generation. The cellular component (CC) category included terms such as condensed chromosome, plasma membrane, nuclear part, chromosome, extracellular matrix, and nuclear lumen. Molecular function (MF) category terms comprised protein kinase activity, lyase activity, ion channel activity, and calcium channel activity (all with *P*-adj < 0.05).

Based on the KEGG pathway enrichment analysis (Fig. S3), the results revealed significant enrichment (*P*-adj < 0.05) of these genes in pathways related to reproductive processes, including aml04912 (GnRH signaling pathway), aml04929 (GnRH secretion), aml04728 (Dopaminergic synapse), aml04024 (cAMP signaling pathway), aml04010 (MAPK signaling pathway), aml04927 (Cortisol synthesis and secretion), aml04726 (Serotonergic synapse), and aml04022 (cGMP-PKG signaling pathway).

### 1.2 Core proteins identified by PPI network

The results of visualization of expression patterns of mating related genes through protein-protein interaction (PPI) network were shown in Figure S4. PPI filtering was conducted by retaining nodes with a minimum required interaction score of 0.4. The PPI enrichment p-value was found to be < 0.001. We further identified ten core proteins (RAF1, MAP2K2, MAP2K1, MAP3K1, NRAS, KRAS, HRAS, SOS1, PIK3CA, and PIK3CD) based on the results of betweenness centrality and degree centrality analyses.

### 1.3 Gene alternative splicing and variant sites

Among the 320 mating related genes, 98 were detected with AS events (Table S8), with skipped exon (SE) being the most frequent type observed in 96 genes, followed by and alternative 3' splice site (A3SS) in 11 genes, alternative 5' splice site (A5SS) in 8 genes, mutually exclusive exon (MXE) in 26 genes, and retained intron (RI) in 9 genes. Eleven genes exhibited three or more AS events, while *CAMK2D*, *CATSPERG*, and *ADCY7* had four AS events. In addition, 146 VS were identified, including 99 genes with InDels events and 140 genes with SNPs events (Table S8).
